# Natural history traits influence winners and losers for herpetological communities in disturbed tropical habitats

**DOI:** 10.1007/s00442-025-05691-7

**Published:** 2025-03-12

**Authors:** Rowland K. Griffin, Todd R. Lewis, Joseph Tzanopoulos, Richard A. Griffiths

**Affiliations:** 1 Durrell Institute of Conservation and Ecology, School of Anthropology and Conservation, University of Kent, Marlowe Building, Canterbury, Kent CT2 7NR UK; 2 Department of Conservation, La Aurora National Zoo, Guatemala City, Guatemala; 3 UWE Bristol – Frenchay Campus, Coldharbour Ln, Bristol, BS16 1QY UK

**Keywords:** Functional ecology, Reptile, Amphibian, Change in land-use, GLLVM, Winner/loser replacements

## Abstract

Habitat alteration can lead to a few ‘winning’ species outcompeting many ‘losing’ species, an effect commonly termed as ‘Winner-Loser-Replacements’ or WLRs. This can lead to homogenisation of species assemblages at phylogenetic and functional levels. Most previous studies analyse responses of species abundance without considering natural history traits associated with those species. This study uses fourth corner modelling techniques to investigate the interaction between ecological data and natural history trait information using a herpetofaunal assemblage that includes 19 species of amphibians, 28 snakes, and 20 lizards, in Parque Nacional Laguna del Tigre, Guatemala. A total of 120 transects were surveyed using Visual Encounter Surveys, comprising 18 in disturbed habitat, 66 in forest habitat, and 36 in edge habitat respectively. Overall, greater diversity of ecological traits was revealed in forest and edge habitats compared to disturbed habitats at the forest edge close to agricultural land. Models revealed that for amphibians (*Hypopachus variolosus* and *Incilius valliceps*) and snakes (*Coniophanes schmidtii* and *Leptodeira septentrionalis*), association with bare ground, and in the case of amphibians, leaf litter, predicts species persistence in disturbed habitats. Continued forest fragmentation in the region will result in increased edge effects, and a greater proportion of forest remaining in an early successional state, leading to a highly reduced, homogenized, amphibian and reptile assemblage. Using such models for community assemblages of animals to reveal the identity of WLR patterns in forests with continued fragmentation is a useful tool to reveal which species are at risk of impact before habitats become degraded.

**Supplementary Information:**

The online version contains supplementary material available at 10.1007/s00442-025-05691-7.

## Introduction

Habitat alteration is one of the most widespread causes of biodiversity loss, directly affecting the species occurring in the altered habitat, as well as indirectly affecting species in adjacent intact habitat (Laurance 2008). The loss of natural habitat resulting from a change in land use often creates unnatural forest edges. In tropical forests, the vegetation of so-called ‘edge habitat’ is often kept in an early successional state, with a highly altered habitat structure and reduced diversity of species, trait diversity, and ecosystem functions (Taberelli and Lopes 2008; Pütz et al. 2011). This reduction in diversity is often driven by increases of native generalist species rather than by invasion of non-natives, through Winner-Loser Replacements, or WLRs, where so called ‘losing’ species are replaced by ‘winning’ species (Tabarelli et al. 2012). However, in some instances replacements can be sufficient to maintain species diversity but with a highly altered assemblage composition (Palmeirim et al. 2017). Losing species tend to exhibit traits such as large size, low fecundity, limited geographical ranges and specialized ecology, combined with low dispersal rates, and poor adaption to human disturbance. Conversely, winning species tend to be widespread generalists with high fecundity and rapid dispersal rates that are well adapted to human disturbance (Tabarelli et al. 2012). Globally, over 50% of all species could be considered losers that are adversely affected by human activity compared to 5 to 29% of native species considered to be winners with either stable or expanding ranges (McKinney and Lockwood 1999). A further 1–2% of species could be considered non-native invasive winners. Therefore, there is an overall effect of replacing many losing species with a few winning species. This leads to assemblage homogenization of genetic and functional levels at both local and global scales (McKinney and Lockwood 1999; Olden et al. 2004; Newbold et al. 2018).

Global meta-analyses indicate that homogenization is occurring globally and across taxonomic groups (McKinney and Lockwood 1999; Newbold et al. 2018). However, the process is currently more pronounced in the tropics (Newbold et al. 2018). This is thought to be due to: (1) distributional ranges that are smaller than the global average; (2) a higher degree of ecological specialization; and (3) that temperate zones have already experienced the large-scale homogenization that the tropics are currently experiencing (Newbold et al. 2018). The homogenizing effect of WLRs has been recorded in invertebrates (Oliveira et al. 2016; Mangels et al. 2017; Filgueirasa et al. 2019), frogs (Cunha Bitar et al. 2015), birds (Villegas Vallejos et al. 2016), mammals (Palmeirim et al. 2020), and plants (Tabarelli et al. 2012; Leal et al. 2015). To date, modelling techniques have not taken trait data into account. Natural history traits and how they interact with environmental parameters and influence species abundance can inform conservation management. The interaction between ecological and trait data is termed the fourth corner (Brown et al. 2014), the advent of fourth corner techniques allows the modelling of abundance data in relation to observed ecological data and recorded trait data, as well as abundance as a function of the interaction between ecological and trait data. Recently, it has been recognized that not only are trait data essential for informing the conservation of species, but also that there is a paucity of trait data available for amphibians and reptiles (Etard et al. 2020).

Populations of amphibians and reptiles are in global decline and are considered among the most threatened vertebrate taxa (Gibbons et al. 2000; Collins and Storfer 2003). Understanding the dynamics of amphibian and reptile declines is hampered as many species undergo natural fluctuations in populations that often require long-term data collected over decades to identify population trends (Pechmann et al. 1991; Alford and Richards 1999; Whitfield et al. 2007). The diversity of amphibian and reptile species is highest in the tropics, as are the level of threats to their populations and habitats (Vitt and Caldwell 2014; Newbold et al. 2018). Several studies have identified WLRs in tropical amphibian and reptile assemblages in response to anthropogenic pressures (Gallmetzer and Schulze 2015; Hirschfeld and Rödel 2017; Nowakowski et al. 2018). This study extends these previous studies by utilizing fourth corner modelling techniques to identify WLRs between amphibian and reptile species, the environment, and their traits. Using such models to study animal communities in fragmented forests can help identify the risk of species loss through WLR patterns, revealing those species at risk in the community before habitats deteriorate. To the best of our knowledge this is first time this approach has been used in a full amphibian and reptile assemblage. This paper seeks to investigate how natural history traits influence the ability of amphibians and reptiles to successfully exploit disturbed tropical habitats. More precisely, we test the following hypotheses: (1) amphibians and reptiles that live in disturbed tropical habitats have identifiable natural history traits that enable them to successfully exploit disturbed tropical habitats; and (2) amphibians and reptiles behave in specific ways that enable them to successfully exploit disturbed tropical habitats. We predict that species that occur in multiple habitat types are better at adapting to novel and disturbed habitats.

This study used population abundance data collected from a community of amphibians and reptiles with guild factors from known life history information. Combined, the data created a case study to test the interaction of guild traits with population and habitats. A single site in Guatemala, Estación Biológica las Guacamayas (EBG), was chosen for investigation due to its range of both disturbed and undisturbed habitats serving the whole reptile and amphibian community.

## Methods

### Study site

**Fig. 1:**
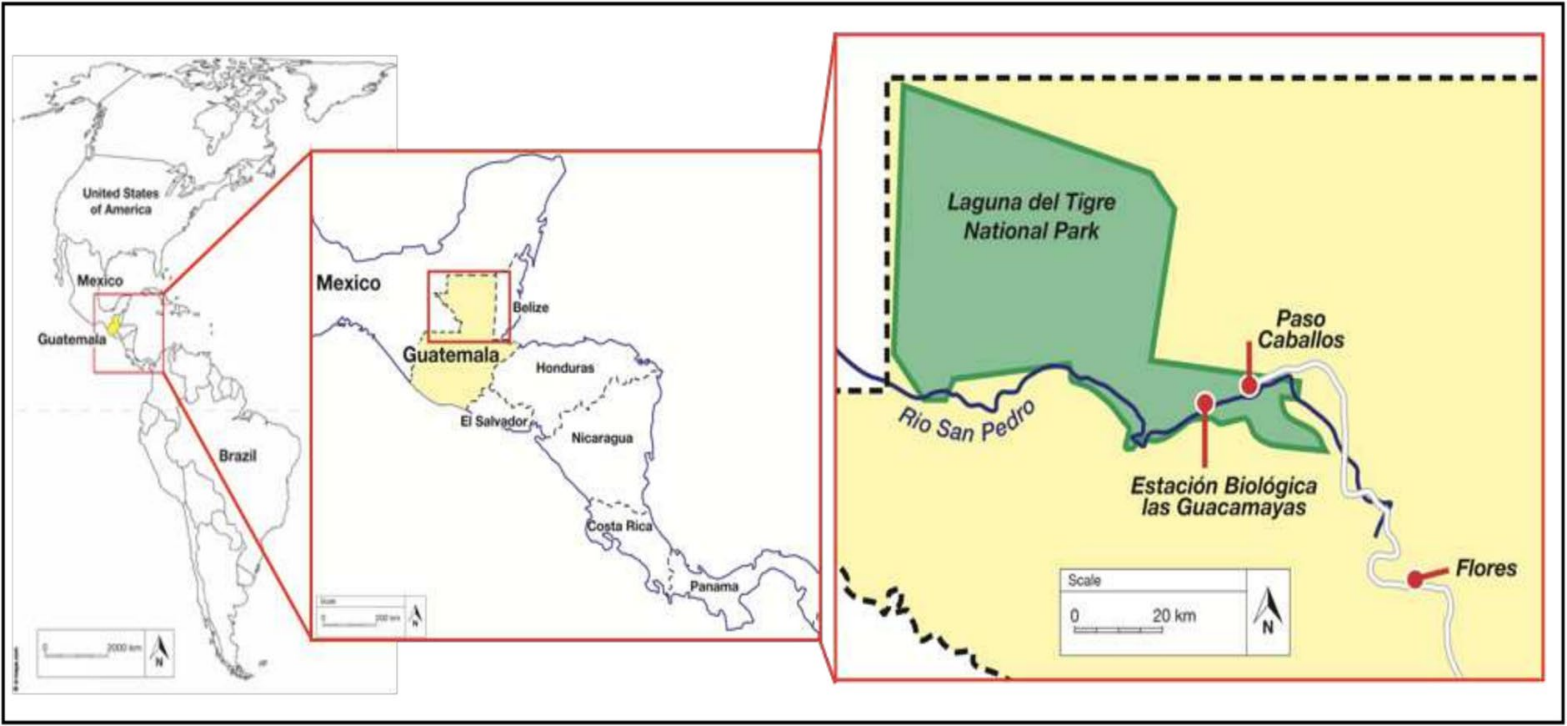
Map of the Americas, showing the location of Parque Nacional Laguna del Tigre within Guatemala. Red box indicates area shown in Fig. 2. Due to the curvature of the map the scale shown is representative of the scale at the equator. Map adapted from D-Maps.com

Estación Biológica las Guacamayas is located in the south-east of Parque Nacional Laguna del Tigre (PNLT) on the banks of the Rio San Pedro (Fig. 1). The Tropical Moist Forest (Holdridge 1967) of EBG consists of several habitat types including both primary and secondary forest, saw-grass swamp and thorn scrub. It is bordered to the east by concessional agricultural lands that belong to the nearby Q’eqchi’ Maya community of Paso Caballos. The work extends on Griffin (2022 unpublished thesis) and Griffin et al. (2023).

### Field methods

**Fig. 2:**
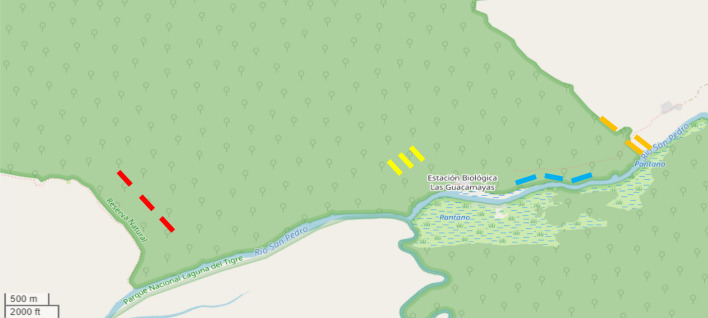
Map of the south-east region of Parque Nacional Laguna del Tigre showing the location of transects indicated by coloured lines: Orange = Disturbed Habitat MH1; Yellow and Red = Forest Habitat MH2; Blue = Edge Habitat MH3. The two rivers are the San Pedro River flowing east to west, and the Sacluc River flowing south to north. North of the San Pedro dark green areas indicate forest areas, the grey in the top right corner indicates the concessional agricultural land of Paso Caballos. South of the San Pedro, green indicates a mixture of saw grass swamp (sabinal) and seasonally flooded thorn scrub

Sampling was conducted in one disturbed edge habitat (named disturbed habitat and assigned the code MH1), two primary forest habitats (named forest habitat with the code MH2), and one secondary habitat with natural edge (named edge habitat with the code MH3) (Fig. 2). Surveys in disturbed habitat (MH1) were conducted at the eastern border with the concessional agricultural lands of the Paso Caballos approximately 2 km east of EBG. This area has been subject to relatively high levels of disturbance from the clearing activities related to the concessions and is considered secondary forest. Forest habitat (MH2) surveys were conducted in two undisturbed forest locations and consisted of mature humid tropical forest that is characterized by relatively low canopy (ca. 25–35 m), well-developed leaf litter with shallow soils, and sparse distribution of standing water bodies. In an attempt to increase detection to permissible levels in order to capture a broad range of species without causing over-parameterization of the models data were collected at two locations within this habitat and subsequently pooled. Surveys in edge habitat (MH3) were conducted in secondary growth forest found between 50 and 100 m from top of a steep limestone cliff that rises from the northern banks of the San Pedro river. Up until 30 years previously this area had been used for maize cultivation.

In each of the four survey locations, three 100 m transects were conducted both along existing trail systems and on transects cut sensitively into the forest away from the trails (Fig. 2). Transects in edge habitats were positioned parallel to the edge such that any edge effects were of consistent strength throughout the length of the transect. Transects were placed to allow a representative sample of each habitat and promote heterogeneous sampling across microhabitats for efficient detection of herpetofauna (Crump and Scott 1994; Doan 2003; Marsh and Haywood 2010). The start points for each transect were chosen to allow for any edge effects to be accounted for that may have risked biasing detection (Schlaepfer and Gavin 2001; Urbina-Cardona et al. 2006). Transects were marked every 25 m with flagging tape to indicate the path of the transect, and GPS waypoints were taken at the start and finish points using a handheld GPS device (Garmin™ GPSMap 62s) to facilitate accurate survey replication. After setup, transects were left for a minimum of two days before surveying commenced to allow for animals to resume normal activity prior to survey (Crump and Scott 1994). All transects had negligible changes in altitude and were positioned so that no habitat transitions occurred during the length of the transect (Babbitt et al. 2009). Surveys took approximately 45 min to one hour to complete and followed standardized protocols for Visual Encounter Surveys in tropical habitats (Rödel and Ernst 2004; Vonesh et al. 2009).

To maximize chances of detecting species with different autecology, each transect was surveyed three times, twice at night and once in the morning during each survey period (Heyer et al. 1994; McDiarmid et al. 2012). A minimum of two days was left between surveys of the same transect to maintain independence of sample survey periods (Doan 2003). Surveys were conducted during seven fieldwork periods in May–June 2013, November–December 2013, June 2014, October 2014, June 2015, December 2015, and June-July 2016. A total of 120 transects were surveyed, comprising 18 in disturbed habitat (MH1), 66 in forest habitat (MH2), and 36 in edge habitat (MH3) respectively. The order in which the three forest habitats were surveyed was randomized, as was the order of transects within each habitat. On a total of 44 survey days (132 transect surveys) fieldwork was hampered by inclement weather, such as incoming hurricanes and severe thunderstorms, and surveys had to be abandoned due to safety concerns and were removed from the dataset prior to analyses, hence the uneven number of surveys between each habitat.

Upon location of an amphibian or reptile the following data were recorded: time encountered (24 h), location (recorded using a Garmin GPSmap 62s), species, microhabitat (aquatic, aquatic margin, bare ground, leaf litter, leaf, tree limb, and tree trunk), behaviour at time of first observation (active, ambush, amplexus [amphibians only], calling [amphibians only], feeding, and stationary). For the purposes of this study, we define ‘ambush’ as a stationary but alert state of being, compared to ‘stationary’ position where there is not obvious intent of forthcoming action. If safe and practical to do so, individuals were captured to confirm identification when needed; if a positive species identification was not possible the individual was excluded from the dataset. Natural history traits were also recorded from the literature (Lee 1996) and included: diel activity patterns (diurnal, nocturnal, active both nocturnally and diurnally, and in the case of diurnal lizards, thermoconformer or heliophilic), prey preference, and body mass categorized into ranges appropriate for each taxon.

Visual encounter surveys are a regularly used method for surveying amphibians and reptiles (Crump and Scott 1994; Lovich et al. 2012). Survey teams were trained and organized to minimize any impacts of surveyor bias (Griffin et al. 2023). Transects were walked at a suitably slow pace to allow detection of reptiles and amphibians by thorough examination of vegetation and refugia, such as leaf litter, fallen limbs and rocks (Crump and Scott 1994; Lovich et al. 2012). The search area was defined as up to one metre each side of the transect and up to 2 m high (Crump and Scott 1994; Lovich et al. 2012). Any individual found outside of this area was recorded as a casual observation but omitted from the analysis.

### Statistical analysis

Guilds for the amphibian and reptile assemblages were assigned following Duellman (2005) as appropriate and based on diel activity, microhabitat preferences, and diet. The data used to form the guilds were derived from a combination of field observations and information contained in Lee (1996). Guilds were employed in this study as they provide a functional framework to group species with similar ecological roles, facilitating comparisons across assemblages and enhancing the interpretation of their responses to environmental factors. By incorporating guilds as well as individual species, the approach accounts for ecological redundancy and highlights broader patterns in habitat selection and natural history traits.

Generalized Linear Latent Variable Modelling (GLLVM) was performed using package gllvm in R (ver. 4.0.2; Niku et al. 2019a; R Core Team 2021) to analyse factors influencing the amphibian and reptile assemblages in PNLT and to compare species-specific responses to forest habitat selection and natural history traits. GLLVM extends traditional generalized linear models to multivariate data by incorporating latent variables, which serve as ordination axes that model correlations between responses through factor loadings. These latent variables can predict new values, control for known variables, and assist in model selection (Hui et al. 2015, 2017). By integrating environmental predictors with species traits, a trait covariate model, also referred to as a fourth corner model, can be constructed (Brown et al. 2014; Warton et al. 2015). Separate models were developed for amphibians, snakes, and lizard assemblages. For statistical analysis, nocturnal and diurnal survey data were pooled.

Each model incorporated three matrices:

1. Abundance matrix (response variable): Species abundance data for each forest microhabitat category, where abundance was defined as the total number of individuals observed across all transects within a habitat. To identify if the response of a species changes depending on which microhabitat it was found in species were separated accordingly, i.e., if species A was found in MH1 and MH2 habitats then those abundance data were identified in the following way ‘speciesA_MH1’ and ‘speciesA_MH2’.
2. Environment matrix (predictor variable): Frequency of habitat usage and observed behaviours (Table 1).
3. Trait matrix (fourth corner): Functional traits for each species within a microhabitat, derived from a combination of observed traits and known traits from literature sources (e.g., Lee 1996). These traits included diel activity, foraging mode (amphibians were all classified as sit-and-wait predators), prey consumption (snakes only), prey preferences, and body mass (Table 2).

**Table 1:** Categories used for variables in the Environmental Matrix for GLLVM models of the amphibian, snake, and lizard assemblages of PNLT

Environmental variable	Model categories		
	Amphibians	Snakes	Lizards
Microhabitat	Aquatic	Aquatic	Bare ground
	Bare ground	Bare ground	Leaf litter
	Leaf litter	Leaf litter	Tree limb
	Tree limb	Logs	Tree trunk
	Tree trunk	Tree limb	
		Tree trunk	
Activity	Active	Active	Active
	Ambush	Ambush	Ambush
	Amplexus	Feeding	Basking
	Calling	Stationary	Stationary
	Feeding		
	Stationary

**Table 2:** Categories used for variables in the Trait Matrix for GLLVM models of the amphibian, snake, and lizard assemblages of PNLT

Trait variable	Model categories		
	Amphibians	Snakes	Lizards
Diel activity	Nocturnal	Nocturnal	Diurnal thermoconformer
	Diurnal and nocturnal	Diurnal and nocturnal	Diurnal heliothermic
		Diurnal	Nocturnal
Prey preference	Ants	Amphibians	Arachnids
	Insects	Birds	Insects
	Fish	Earthworms	Larval insects
	Frogs	Mammals	Small insects
	Mammals	Snakes	Frogs
	Termites	Lizards	Fruit
		Snails	Lizards
		Fish	
		Reptile eggs	
Mass	< 2 g	< 5 g	< 2 g
	2–9.9 g	5–25 g	2–5 g
	10–29.9 g	26–50 g	10–20 g
	30–100 g	101–1000 g	21–50 g
		1001–2000 g	51–100 g
		> 2000g	> 100 g
Foraging mode	Sit and wait	Active	Active
		Sit and wait	Sit and wait
Prey consumption		Constrictor	
		Grab and swallow	
		Venom	

The GLLVM regressed mean species abundances against environmental predictors such as microhabitat selection and observed behaviours, with the Abundance and Environment matrices coupled to the Trait Matrix to create a full fourth corner model. Model diagnostics, including Dunn-Smyth residual plots and Q-Q plots, confirmed that models did not exhibit overdispersion (Supplementary material, Figures S.1–6). Negative binomial distributions were used for all models, as they outperformed Poisson distributions in terms of coefficient stability and reduced over-correlation, despite Poisson models occasionally having slightly lower BIC values (Supplementary material, Figures S.7–9).

Model fit was further evaluated using AIC and BIC comparisons, with BIC preferred for its finer resolution. A for-loop was implemented to identify the optimal number of latent variables, selecting the model with the highest log-likelihood from five independent runs (Niku et al. 2019b). Based on BIC values, two latent variables were consistently assigned for all models (Supplementary material, Figures S.10–12). Latent variables induced correlation across response variables, enabling estimation of correlation patterns and their explanation by predictor variables, with the goal of minimizing residual correlation.

To quantify and visualize correlation, the getResidualCor function from gllvm was applied to estimate linear predictor correlations across amphibian and reptile presence, with results visualized using the package corrplot (Wei and Simko 2017). The same function was used to evaluate (co)variation by individual predictors (e.g., natural history traits). Predictor coefficients and their confidence intervals were visualized using the coefplot function in gllvm, providing insight into species-specific relationships with predictor variables. Coefficients for the fourth corner traits were further plotted using the lattice package (Sarkar 2020), to illustrate the interplay between observed behaviours, microhabitat selection, and natural history traits.

Both positive and negative coefficient associations were detected in the models. However, it is important to note that negative associations are often a result of no detection or observation of factor, and their strength can be indicative of a lack of probability of occurrence in the model. Therefore, we concentrate on reporting positive associations since they show a species preference and/or association to a given factor.

## Results

A total of 745 individuals (411 amphibians, 174 snakes, 160 lizards) were observed during the study, consisting of 66 species (19 amphibians, 28 snakes, 19 lizards). 18 species were recorded in MH1, 56 species in MH2, and 41 in MH3. A total of 33 species only occurred in one habitat type (7 amphibians, 16 snakes, 10 lizards), 17 occurred in two habitat types (7 amphibians, 6 snakes, 4 lizards), and 16 occurred in all three (5 amphibians, 6 snakes, 5 lizards). Raw data are included in an open access repository for inspection along with model code.

### Amphibian assemblage

**Fig. 3:**
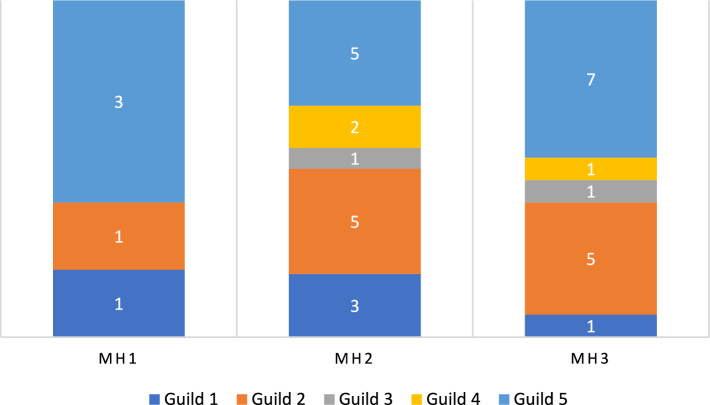
Frequency graph showing the number (white numerals) of amphibian species per guild found in each of the three habitat types: MH1 = Disturbed habitat; MH2 = Forest habitat; MH3 = Edge habitat. Guilds: 1 = Nocturnal, Terrestrial, Ants/termites; 2 = Nocturnal, Terrestrial, other insects; 3 = Nocturnal, Terrestrial, insects/vertebrates; 4 = Nocturnal, aquatic, insects/vertebrates; 5 = Nocturnal, arboreal, insects

**Fig. 4:**
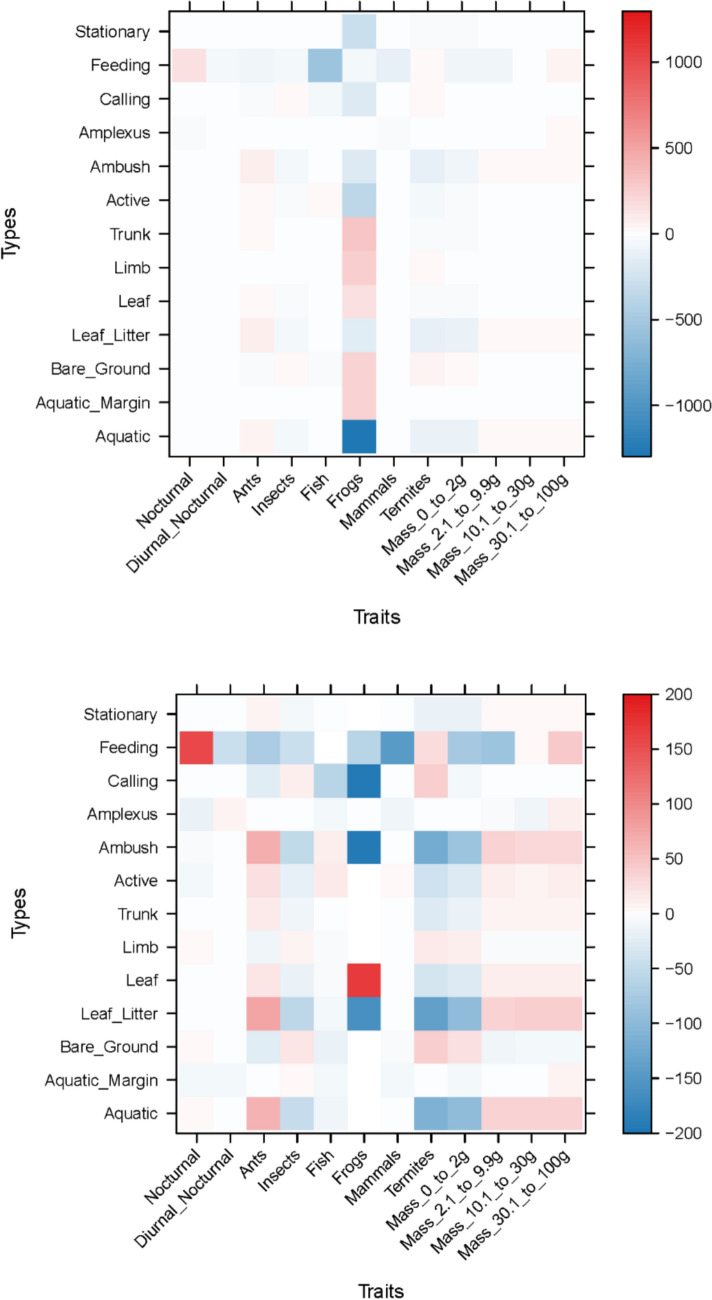
Lattice plots of natural history traits in relation to microhabitat and behavioural observation in the amphibian assemblage of Parque Nacional Laguna del Tigre. The upper plot shows relationships with coefficient values up to 2000, the lower shows the relationships with sensitivity reduced to 200. Red squares indicate significant positive relationships, blue squares indicate significant negative relationships. The stronger the colour, the stronger the signal

The amphibian assemblage was categorized into five guilds using published data contained in Lee (1996; Table 3 and Fig. 3). The lattice plot produced for the trait model showed slight positive signals between ants and leaf litter, and termites and bare ground (Fig. 4). Coupled with the negative signal of termites and leaf litter, this could indicate different foraging strategies in Guild 1 (Table 3). Species that feed on frogs show a strong association with aquatic microhabitats. Two microhabitats were shown to be particularly important for amphibians, aquatic margins and microhabitats associated with arboreality. This suggests that the amphibian assemblage in PNLT can be broadly described in two major groupings, terrestrial species that congregate at the water’s edge, and arboreal species. Since the initial lattice plot did not reveal many associations, we rescaled the plot to increase sensitivity. This revealed further relationships between the observed environmental variables and the natural history traits of amphibians (Fig. 4). Most notably between amphibian species that feed on ants and aquatic and leaf litter microhabitats, and ambush and stationary behaviours.

**Table 3:** Amphibian guilds in Parque Nacional Laguna del Tigre, Guatemala

Guild number	Guild description	Species	Family
1	Nocturnal, terrestrial, ants/termites	*Gastrophryne elegans*	Microhylidae
		*Hypopachus variolosus*	Microhylidae
		*Rhinophrynus dorsalis*	Rhynophrynidae
2	Nocturnal, terrestrial, other insects	*Eleutherodactylus leprus*	Eleutherodactylidae
		*Engystomops pustulosus*	Leiuperidae
		*Incilius valliceps*	Bufonidae
		*Leptodactylus fragilis*	Leptodactylidae
		*Leptodactylus melanonotus*	Leptodacylidae
3	Nocturnal, terrestrial, insects/vertebrates	*Rhinella horribilis*	Bufonidae
4	Nocturnal, aquatic, insects/vertebrates	*Lithobates brownorum*	Ranidae
		*Lithobates vailantii*	Ranidae
5	Nocturnal, arboreal, insects	*Bolitoglossa mexicana*	Plethodontidae
		*Agalychnis callidryas*	Hylidae
		*Dendropsophus microcephalus*	Hylidae
		*Scinax staufferi*	Hylidae
		*Smilisca baudinii*	Hylidae
		*Tlalocohyla loquax*	Hylidae
		*Tlalocohyla picta*	Hylidae
		*Trachycephalus vermiculatus*	Hylidae

**Fig. 5:**
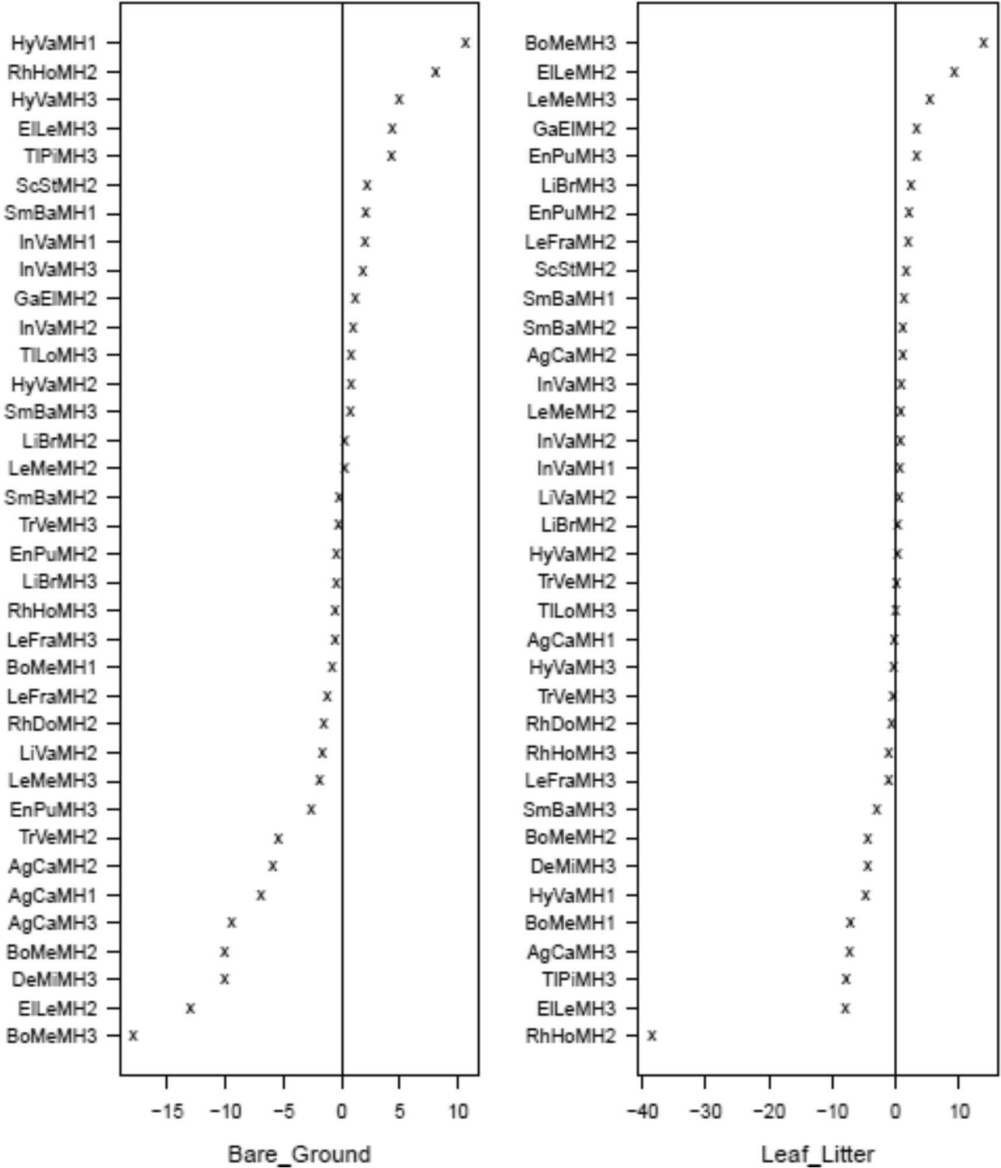
Latent variable coefficient plots showing associations between amphibian species and microhabitats. Significant coefficients are identified by their confidence intervals not crossing zero. Those significant coefficients that are above zero show a positive association to the microhabitats, those that are below zero show a negative association. Habitat codes: MH1 = Disturbed habitat; MH2 = Forest habitat; MH3 = Edge habitat. Species codes: AgCa = *Agalychnis callidryas*; BoMe = *Bolitoglossa mexicana*; DeMi = *Dendropsophus microcephalus*; ElLe = *Eleutherodactylus leprus*; EnPu = *Engystomops pustulosus*; GaEl = *Gastrophyryne elegans*; HyVa = *Hypopachus variolosus*; InVa = *Incilius valliceps*; LeFra = *Leptodactylus fragilis*; LeMe = *Leptodactylus melanonotus*; LiBr = *Lithobates brownorum*; LiVa = *Lithobates vaillanti*; RhDo = *Rhinophrynus dorsalis*; RhHo = *Rhinella horriblis*; ScSt = *Scinax staufferi*; SmBa = *Smilisca baudinii*; TlLo = *Tlalocohyla loquax*; TlPi = *Tlalocohyla picta*; TrPe = *Triprion petasatus*; TrVe = *Trachycephalus vermiculatus*

**Fig. 6:**
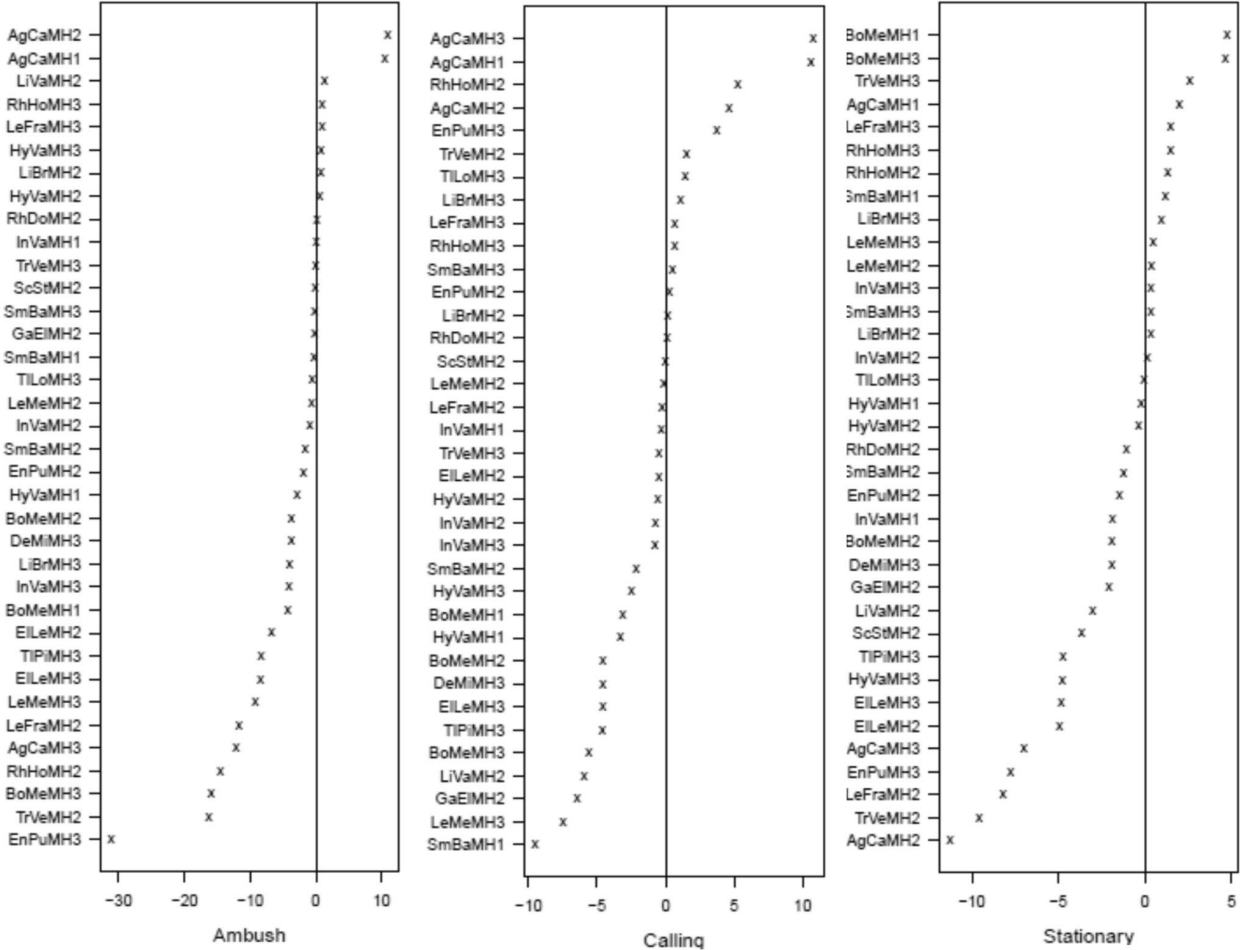
Latent variable coefficient plots showing associations between amphibian species and behaviours. Significant coefficients are identified by their confidence intervals not crossing zero. Those significant coefficients that are above zero show a positive association to the behaviours, those that are below zero show a negative association. Habitat codes: MH1 = Disturbed habitat; MH2 = Forest habitat; MH3 = Edge habitat. Species codes: AgCa = *Agalychnis callidryas*; BoMe = *Bolitoglossa mexicana*; DeMi = *Dendropsophus microcephalus*; ElLe = *Eleutherodactylus leprus*; EnPu = *Engystomops pustulosus*; GaEl = *Gastrophyryne elegans*; HyVa = *Hypopachus variolosus*; InVa = *Incilius valliceps*; LeFra = *Leptodactylus fragilis*; LeMe = *Leptodactylus melanonotus*; LiBr = *Lithobates brownorum*; LiVa = *Lithobates vaillanti*; RhDo = *Rhinophrynus dorsalis*; RhHo = *Rhinella horriblis*; ScSt = *Scinax staufferi*; SmBa = *Smilisca baudinii*; TlLo = *Tlalocohyla loquax*; TlPi = *Tlalocohyla picta*; TrPe = *Triprion petasatus*; TrVe = *Trachycephalus vermiculatus*

Coefficient plots of the latent variable trait model revealed significant associations with all microhabitat categories and behaviours with the exception of feeding behaviour (Figs. 5, 6, and supplementary material Figure S.13). Most amphibian species found in disturbed habitat (MH1) tended to use bare ground or leaf litter (Fig. 5). Of the species occurring in disturbed habitat, only the treefrog *Agalychnis callidryas* showed significant associations with microhabitats related to arboreality. Only two species, *A. callidryas* and *Bolitoglossa mexicanus,* were significantly associated with behaviours (ambush, calling, and stationary) in disturbed habitat (Fig. 6). In forest (MH2) and edge (MH3) habitat amphibians fell into three categories of microhabitat usage, those that utilized aquatic microhabitats, those that prefer drier terrestrial microhabitats such as bare ground and leaf litter, and those that utilize arboreal microhabitats. Most amphibians in forest habitat tended to be active hunters (nine species), although two species associated with ambush strategies (MH1) tended to use bare ground or leaf litter. In both forest and edge habitat amphibians also exhibited behaviours related to breeding (calling and amplexus) potentially due to the presence of water bodies in these habitats. Amphibians were often stationary when encountered in all three habitats.

### Snake assemblage

**Fig. 7:**
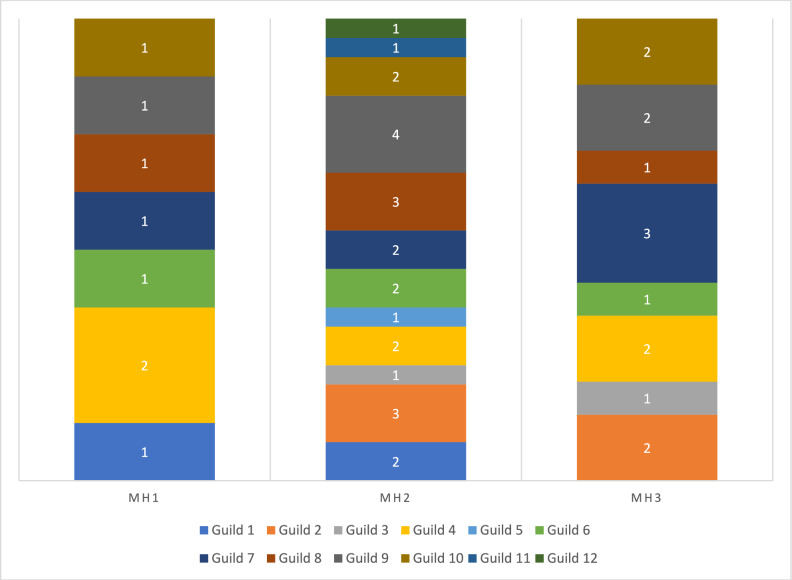
Frequency graph showing the number (white numerals) of snake species per guild found in each of the three habitat types: MH1 = Disturbed habitat; MH2 = Forest habitat; MH3 = Edge habitat. Guilds: 1 = Diurnal, arboreal, lizards and amphibians; 2 = Diurnal, terrestrial, amphibians and lizards; 3 = Diurnal, terrestrial, large, reptiles and mammals; 4 = Nocturnal, arboreal, lizards and amphibians; 5 = Nocturnal, arboreal, birds and mammals; 6 = Nocturnal, arboreal, gastropods; 7 = Nocturnal, terrestrial, lizards and snakes; 8 = Nocturnal, terrestrial, amphibians; 9 = Nocturnal, terrestrial, earthworms and gastropods; 10 = Nocturnal, terrestrial, mammals and birds; 11 = Nocturnal, terrestrial, invertebrates; 12 = Nocturnal, aquatic, fish

**Fig. 8:**
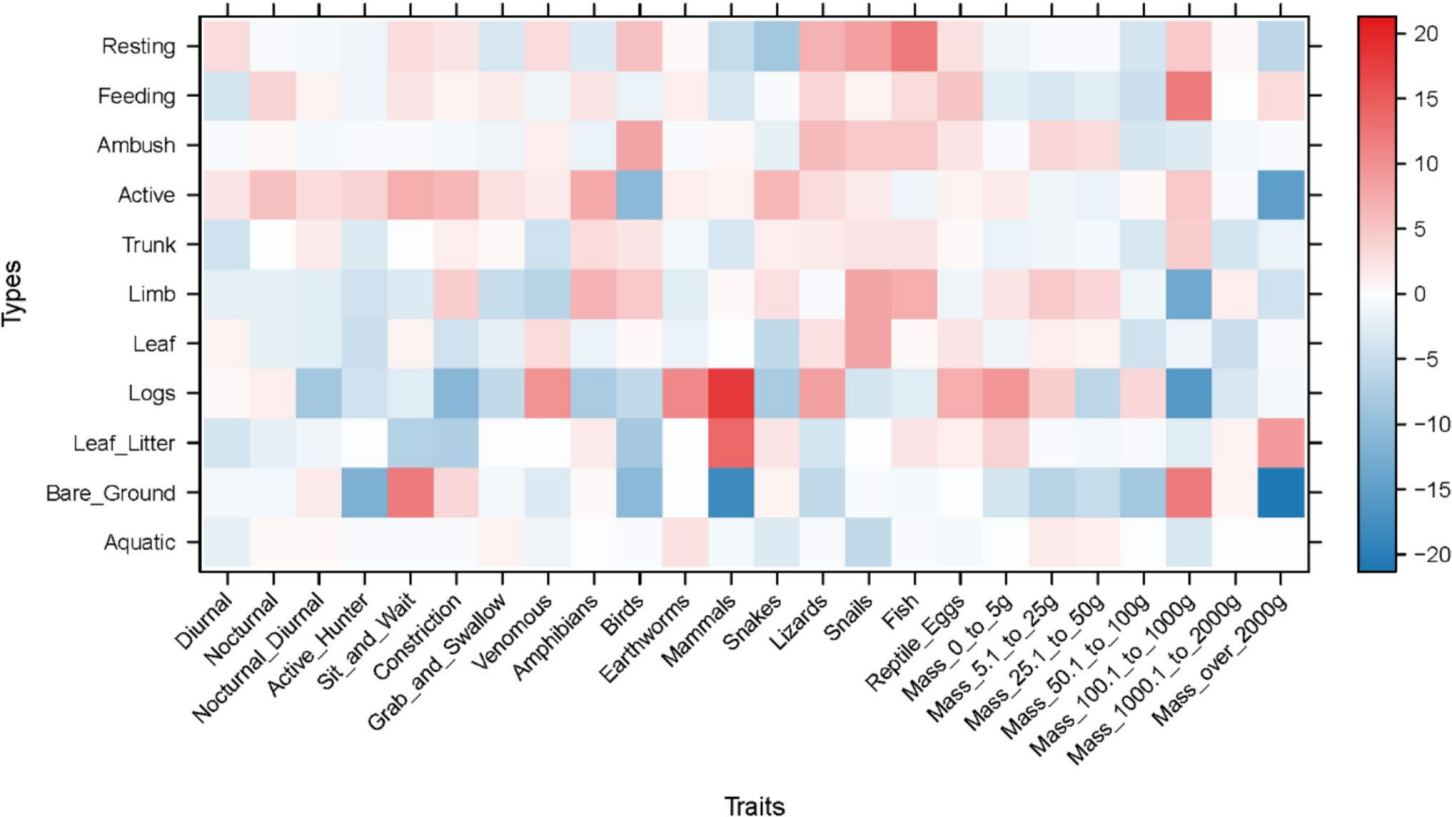
Lattice plot of natural history traits in relation to microhabitat and behavioural observation in the snake assemblage of Parque Nacional Laguna del Tigre. Red squares indicate significant positive relationships, blue squares indicate significant negative relationships. The stronger the colour, the stronger the signal

The snake assemblage was categorized into 12 guilds using published data contained in Lee (1996; Table 4 and Fig. 7). The lattice plot for the snake trait model (Fig. 8) revealed that venomous snakes and snakes that eat earthworms, mammals, and lizards had a strong preference for using logs as a microhabitat. A strong relationship between eating mammals and being an active hunting species was also revealed. Medium strength relationships were revealed between snail-eating species and microhabitat preferences that suggested arboreal tendencies. Additionally, a weak relationship was revealed between snail-eating species and terrestrial habits and between snakes that eat amphibians and arboreal habits. Larger snakes tended to be terrestrial, whereas smaller snakes tend to be arboreal. Rescaling of the lattice trait plot did not reveal further relationships between observed microhabitat selection and behaviour, and natural history traits.

**Table 4:** Snake guilds in Parque Nacional Laguna del Tigre, Guatemala

Guild number	Guild description	Species	Family
1	Diurnal, arboreal, lizards and amphibians	*Leptophis praestans*	Colubridae
		*Leptophis mexicanus*	Colubridae
		*Oxybelis koehleri*	Colubridae
2	Diurnal, terrestrial, amphibians and lizards	*Drymobius margaritiferus*	Colubridae
		*Mastigodryas melanolomus*	Colubridae
		*Xenodon rabdocephalus*	Colubridae
3	Diurnal, terrestrial, large, reptiles and mammals	*Masticophis mentovarius*	Colubridae
		*Spilotes pullatus*	Colubridae
4	Nocturnal, arboreal, lizards and amphibians	*Imantodes cenchoa*	Colubridae
		*Leptodeira septentrionalis*	Colubridae
5	Nocturnal, arboreal, birds and mammals	*Pseudelaphe flavirufa*	Colubridae
6	Nocturnal, arboreal, gastropods	*Sibon dimidiatus*	Colubridae
		*Sibon nebulatus*	Colubridae
7	Nocturnal, terrestrial, lizards and snakes	*Clelia scytalina*	Colubridae
		*Micrurus apiatus*	Elapidae
		*Oxyrhopus petolarius*	Colubridae
8	Nocturnal, terrestrial, amphibians	*Coniophanes imperialis*	Colubridae
		*Coniphanes schmidti*	Colubridae
		*Pliocercus elapoides*	Colubridae
9	Nocturnal, terrestrial, earthworms and gastropods	*Adelphicos quadrivirgattum*	Colubridae
		*Ninia diademata*	Colubridae
		*Ninia sebae*	Colubridae
		*Geophis sartorii*	Colubridae
10	Nocturnal, terrestrial, mammals and birds	*Bothrops asper*	Viperidae
		*Boa imperator*	Boidae
		*Lampropeltis abnorma*	Colubridae
11	Nocturnal, terrestrial, invertebrates	*Tantilla moesta*	Colubridae
12	Nocturnal, aquatic, fish	*Coniophanes bipunctatus*	Colubridae

**Fig. 9:**
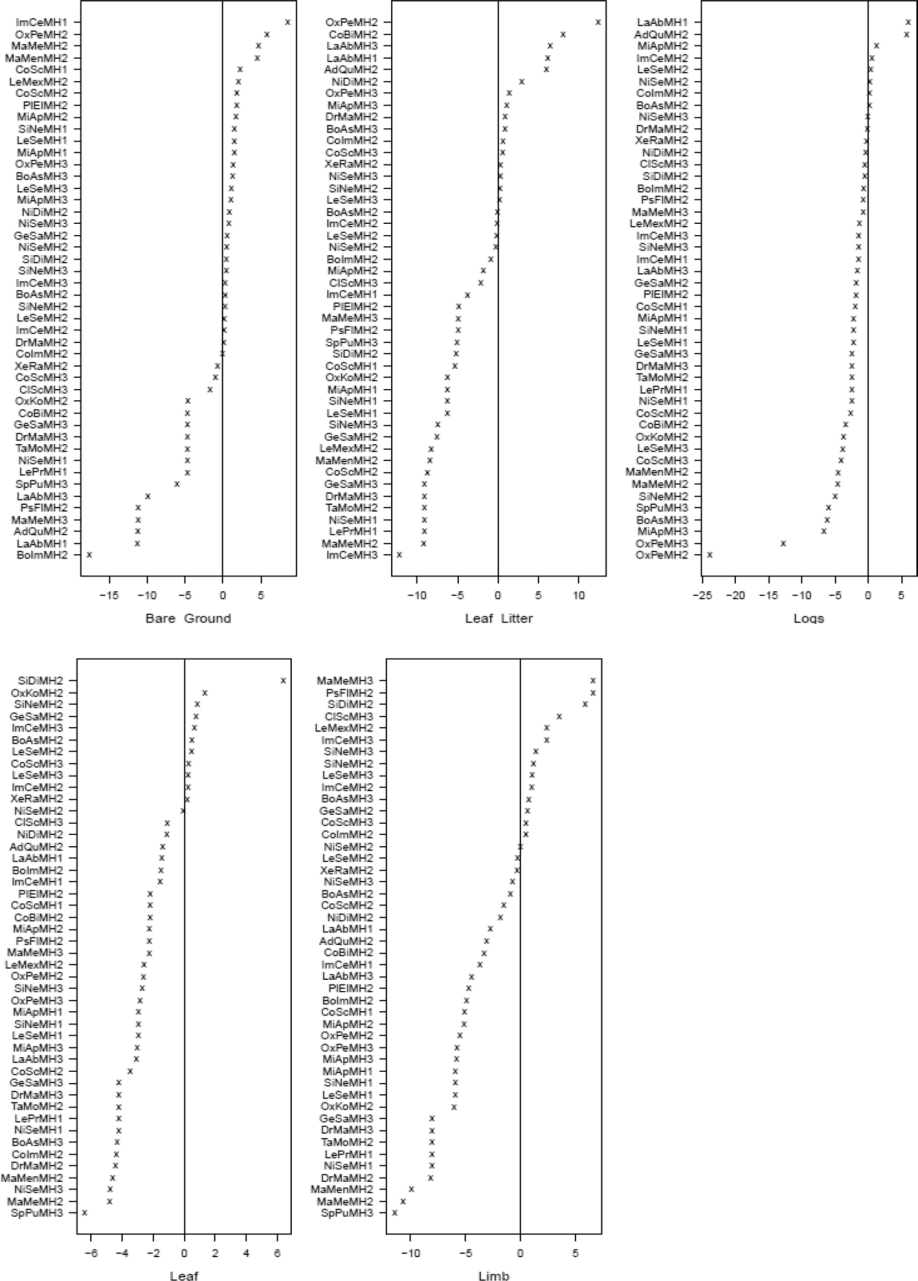
Latent variable coefficient plots showing associations between snake species and microhabitats. Significant coefficients are identified by their confidence intervals not crossing zero. Those significant coefficients that are above zero show a positive association to the microhabitats, those that are below zero show a negative association. Habitat codes: MH1 = Disturbed habitat; MH2 = Forest habitat; MH3 = Edge habitat. Species codes; AdQu = *Adelphicos quadrivigattum*, BoAs = *Bothrops asper*, BoIm = *Boa imperator*, ClSc = *Clelia scytalina*, CoBi = *Coniophanes bipunctatus*, CoIm = *Coniophanes imperialis*, CoSc = *Coniophanes schmidtii*, DrMa = *Drymobius margaritiferus*, GeSa = *Geophis sartorii*, ImCe = *Imantodes cenchoa*, LaAb = *Lampropeltis abnorma*, LeMex = *Leptophis mexicanu*s, LePr = *Leptophis praestans*, LeSe = *Leptodiera septentrionalis*, MaMen = *Masticophis mentovarius*, MaMe = *Mastigodryas melanonomus*, MiAp = *Micrurus apiatus,* NiDi = *Ninia diademata*, NiSe = *Ninia sebae*, OxKo = *Oxybelis koehleri*, OxPe = *Oxyrhopus petolarius*, PlEl = *Pliocercus elapoides*, PsFl = *Psuedelaphe flavirufa*, SiDi = *Sibon dimidiatus*, SiNe = *Sibon nebulatus*, SpPu = *Spilotes pullatus*, TaMo = *Tantilla moesta*, XeRa = *Xenodon rabdocephalus*

**Fig. 10:**
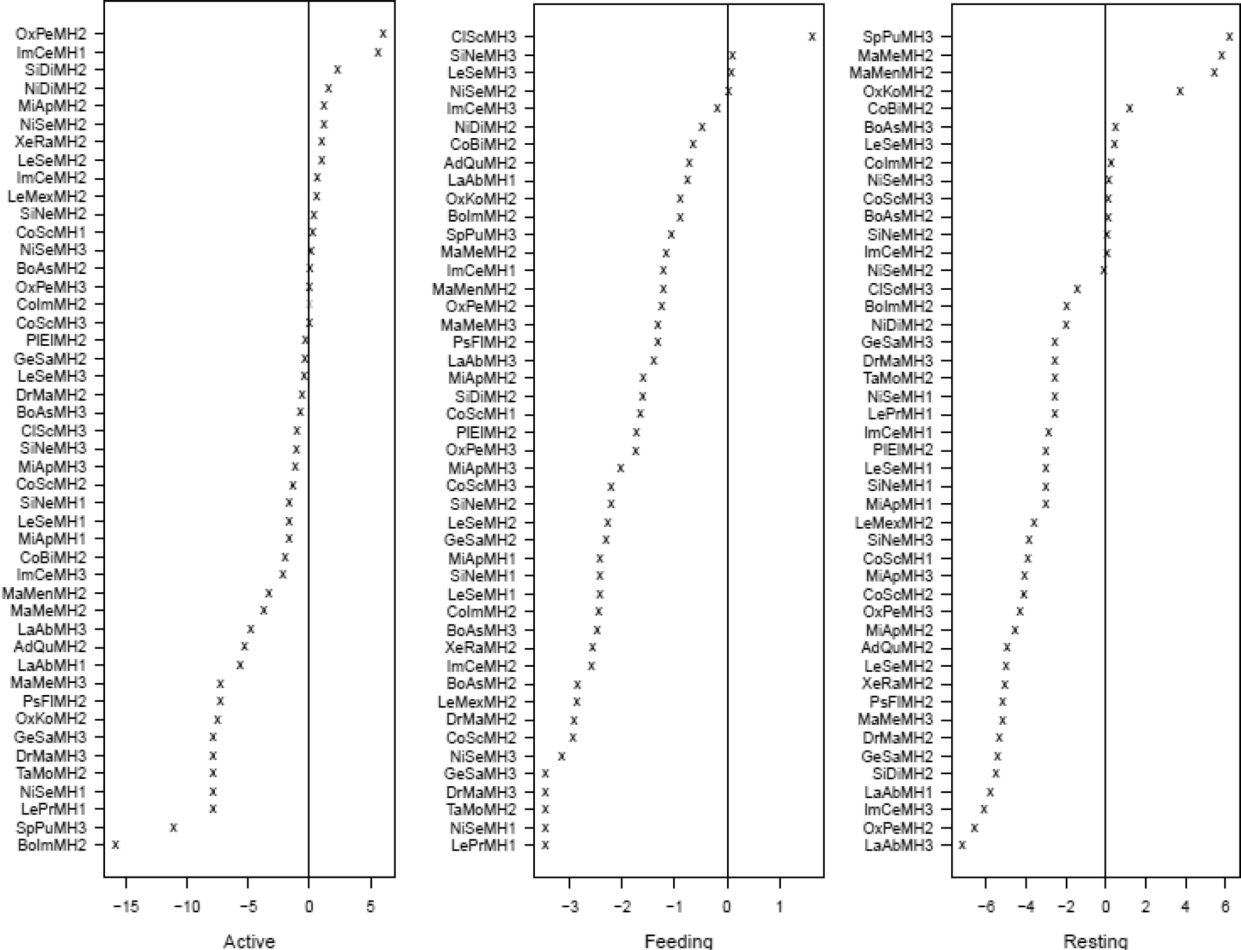
Latent variable coefficient plots showing associations between snake species and behaviours. Significant coefficients are identified by their confidence intervals not crossing zero. Those significant coefficients that are above zero show a positive association to the behaviours, those that are below zero show a negative association. Habitat codes: MH1 = Disturbed habitat; MH2 = Forest habitat; MH3 = Edge habitat. Species codes; AdQu = *Adelphicos quadrivigattum*, BoAs = *Bothrops asper*, BoIm = *Boa imperator*, ClSc = *Clelia scytalina*, CoBi = *Coniophanes bipunctatus*, CoIm = *Coniophanes imperialis*, CoSc = *Coniophanes schmidtii*, DrMa = *Drymobius margaritiferus*, GeSa = *Geophis sartorii*, ImCe = *Imantodes cenchoa*, LaAb = *Lampropeltis abnorma*, LeMex = *Leptophis mexicanu*s, LePr = *Leptophis praestans*, LeSe = *Leptodiera septentrionalis*, MaMen = *Masticophis mentovarius*, MaMe = *Mastigodryas melanonomus*, MiAp = *Micrurus apiatus,* NiDi = *Ninia diademata*, NiSe = *Ninia sebae*, OxKo = *Oxybelis koehleri*, OxPe = *Oxyrhopus petolarius*, PlEl = *Pliocercus elapoides*, PsFl = *Psuedelaphe flavirufa*, SiDi = *Sibon dimidiatus*, SiNe = *Sibon nebulatus*, SpPu = *Spilotes pullatus*, TaMo = *Tantilla moesta*, XeRa = *Xenodon rabdocephalus*

Latent variable coefficient plots revealed significant associations with all forest habitat types (Fig. 9 and supplementary material Figure S.14). In general, a greater diversity of snake natural history traits was found in forest habitat compared to disturbed or edge habitat. Snakes in disturbed habitat (MH1) tended to be associated with terrestrial microhabitats, bare ground, leaf litter, and logs (Fig. 9), although the arboreal snake *Imantodes cenchoa* was associated with leaf and limb (Fig. 9). In disturbed habitat (MH1) snakes tended to be active and feeding when encountered (Fig. 10). Significant associations were found between seven microhabitat categories and snakes found in forest habitat (MH2). Snakes in forest habitat tended to use aquatic, terrestrial, and arboreal microhabitats. They tended to be either active or stationary/in ambush when encountered. Significant associations were found between snake species encountered in edge habitat (MH3) and five microhabitat categories. Snakes in edge habitat tended to be associated with terrestrial and arboreal microhabitats and were commonly encountered feeding and stationary.

### Lizard assemblage

**Fig. 11:**
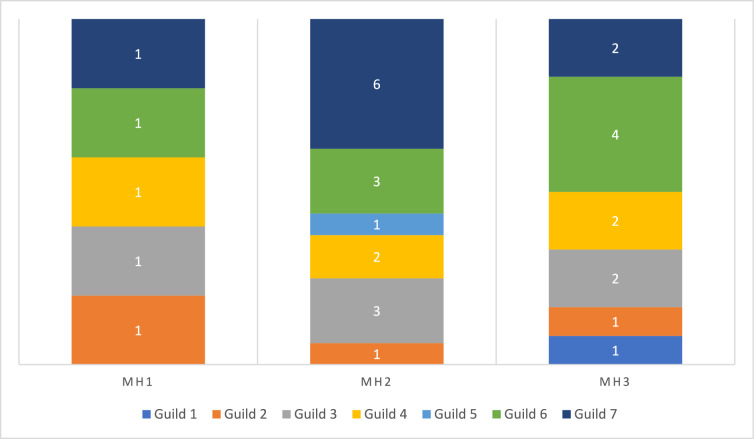
Frequency graph showing the number (white numerals) of lizard species per guild found in each of the three habitat types: MH1 = Disturbed habitat; MH2 = Forest habitat; MH3 = Edge habitat. Guilds: 1 = Nocturnal, arboreal, large insects; 2 = Nocturnal, terrestrial, small, arachnids; 3 = Diurnal, arboreal, large, insects; 4 = Diurnal, arboreal, medium, insects; 5 = Diurnal, bush, small, insects; 6 = Diurnal, terrestrial, med to large, insects; 7 = Diurnal, terrestrial, small, insects

**Fig. 12:**
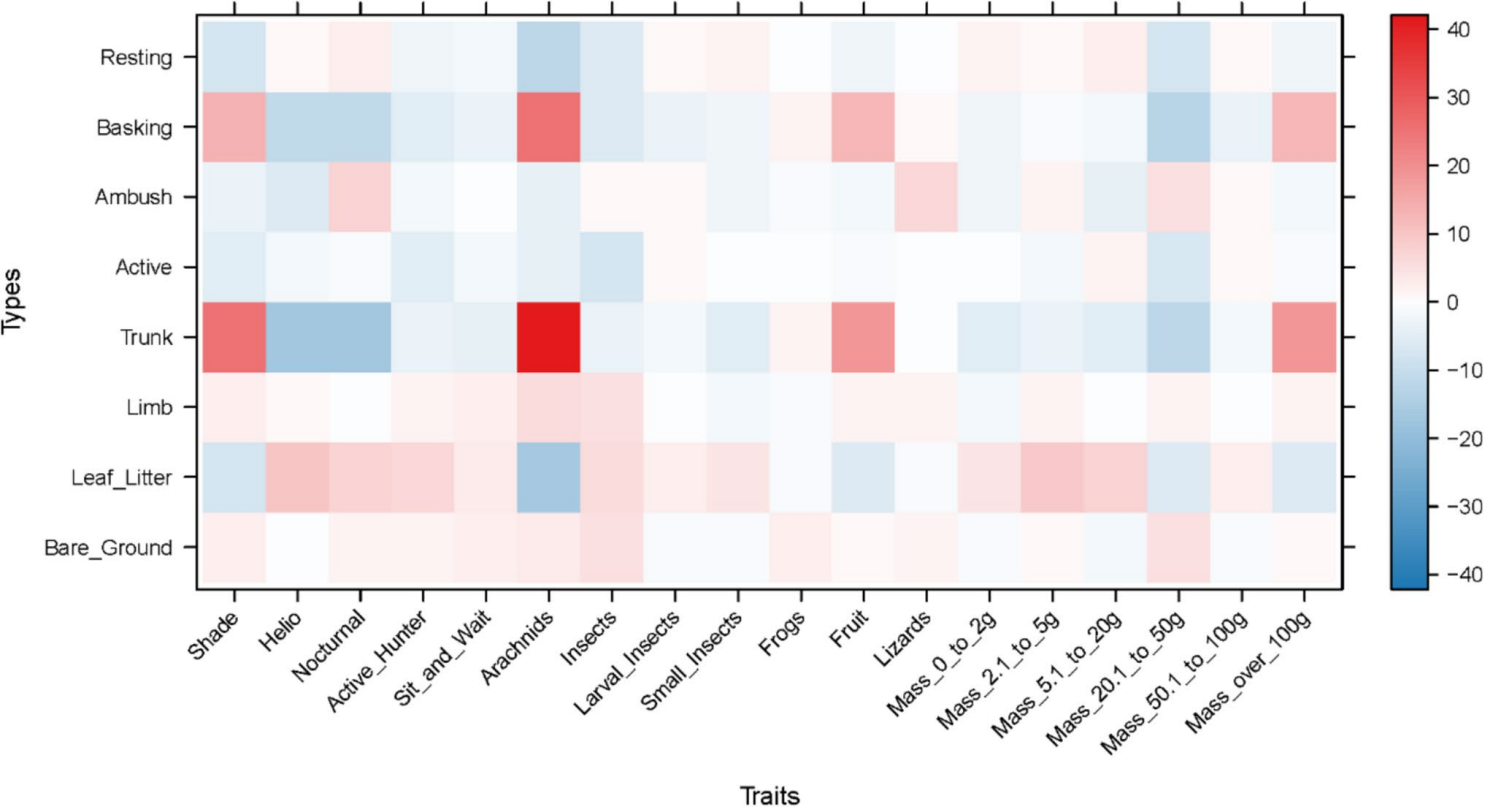
Lattice plot of natural history traits in relation to microhabitat and behavioural observation in the lizard assemblage of Parque Nacional Laguna del Tigre. Red squares indicate significant positive relationships, blue squares indicate significant negative relationships. The stronger the colour, the stronger the signal

Seven lizard guilds were identified using published data contained in Lee (1996; Table 5 and Fig. 11). The lattice plot produced for the trait model (Fig. 12) showed a slight signal between lizard-eating species and ambush behaviour. Moderate signals were found between thermoconformer species and basking on tree trunks, and between heliothermic species and leaflitter. Finally, strong signals were found between species that specialize on eating arachnids and both tree trunks and basking behaviour. Rescaling of the lattice trait plot did not reveal further relationships between observed microhabitat selection and behaviour, and natural history traits. Coefficient plots from the latent variable GLM showed that terrestrial species associate with terrestrial habitats and arboreal species tend to associate with arboreal habitats. However, these associations failed to reveal trends within members of the same guilds, or patterns associated with success in inhabiting a particular habitat (Supplementary material Figure S.15).

**Table 5:** Lizard guilds in Parque Nacional Laguna del Tigre, Guatemala

Guild number	Guild description	Species	Family
1	Nocturnal, arboreal, large insects	*Thecadactylus rapicauda*	Gekkonidae
2	Nocturnal, terrestrial, small, arachnids	*Coleonyx elegans*	Eublepharidae
3	Diurnal, arboreal, large, insects	*Basilicus vittatus*	Corytophanidae
		*Corytophanes cristatus*	Corytophanidae
		*Corytophanes hernandesii*	Corytophanidae
4	Diurnal, arboreal, medium, insects	*Norops capito*	Dactyloidae
		*Norops lemurinus*	Dactyloidae
5	Diurnal, bush, small, insects	*Norops rodriguezii*	Dactyloidae
		*Norops welbornae*	Dactyloidae
6	Diurnal, terrestrial, med to large, insects	*Holcosus festiva*	Teiidae
		*Holcosus undulata*	Teiidae
		*Marisora bracypoda*	Scincidae
		*Mesoscincus schwartzei*	Scincidae
		*Sceloporus teapensis*	Phrynosomatidae
7	Diurnal, terrestrial, small, insects	*Norops tropidonotus*	Dactyloidae
		*Norops unilobatus*	Dactyloidae
		*Sceloporus chrysostictus*	Phrynosomatidae
		*Sphaerodactylus glaucus*	Gekkonidae
		*Sphaerodactylus millepunctatus*	Gekkonidae
		*Scincella cherriei*	Scincidae

## Discussion

Globally, widespread habitat loss is leading to dramatic homogenization of faunal communities and loss of species diversity (McKinney and Lockwood 1999; Laurance 2008; Newbold et al. 2018). This is of particular concern in the tropics where levels of species endemism and ecological specialization is high (Newbold et al. 2018). Understanding the effects of habitat loss, including habitat degradation is essential to aid species conservation. Herein, we provide evidence of how fourth corner modelling can be utilized to elucidate how a species assemblage might respond to such anthropogenic changes, in this case a neotropical herpetofaunal assemblage was used to model how natural history traits might influence those responses.

We were able to use fourth corner GLLVM models to support our hypotheses and identify natural history traits and behaviours in both amphibians and reptiles that are responsible for their successful exploitation of disturbed habitats. GLLVM has been successfully applied to ecological studies investigating cause and effect relations in a model-based framework (Lewis et al. 2021; Rice et al. 2022). Its use as a special case of structural equation model for trait analysis is less applied. To the best of our knowledge this study is the first of its kind to apply selected guilds of an animal group to both population and habitat matrices, in a fourth corner model. The use of a fourth corner or trait model that combines a triple matrix of data provides a unique solution to combining all forms of ecological variables (guild, habitat, morphological, behavioural and abiotic) to interact with each other under a single consensual model applied to abundance data. A major advantage of this method is that once variables have been nested into population, environmental, and trait matrices, the interactions between grouped variables can be controlled hierarchically and variables that suffer mean–variance, or that influence over-dispersion can be easily identified, controlled for, and or selected out (Warton et al. 2016; Niku et al. 2019a). A further advantage is that for more patchy data this method could also be applied in a Bayesian framework (Hui 2016). Our application of this method to macrohabitat and guild is especially novel and we believe could serve as a template for other studies seeking to combine such datasets under a single consensual model framework.

Overall, this study identified a greater diversity of ecological traits in forest and edge habitats compared to the disturbed habitat at the edge of the forest close to the agricultural activities of the Paso Caballos community. This is consistent with other studies of the effects of forest edges and trait diversity (Vallan et al. 2004; Hirshfeld and Rödel 2017). Winning species encountered in disturbed habitats tend to be associated with more terrestrial traits. For example, amphibians are primarily terrestrial and associated with bare ground, and snakes are primarily terrestrial. Whereas, species that associated with arboreal microhabitats tended to be losing species in disturbed habitats. The models failed to identify any associations between lizards, traits, or habitats.

Amphibian species that have significant or near significant associations with bare ground and / or leaf litter in one or more forest habitats are more likely to be found in disturbed habitat where the vegetation is less dense in the other two habitat types. Winning amphibian species in disturbed habitat are represented by three guilds (nocturnal, terrestrial ant specialists; nocturnal, terrestrial insect generalists; and nocturnal, arboreal insect generalists). Terrestrial amphibians seem to group into those that associate with water and those that associate with bare ground/leaf litter. This could explain why *Leptodactylus* species show significant association with leaf litter but are absent from disturbed habitat where water bodies are also absent. The two species of *Leptodactylus* present in PNLT lay foam nests on the surface of ephemeral pools, as such their local distribution in the park is tied closely to the presence of this resource. Several *Leptodactylus* species have been shown to associate strongly with aquatic habitats in natural and agricultural habitats (Lee 1996; Souza et al. 2014). This suggests that the presence, or absence, of water plays an important role in the amphibian assemblage, as does the ability to tolerate open, drier habitats.

Lattice plots revealed separation within amphibian Guild 1 (nocturnal, terrestrial, ant/termite eaters). Feeding on ants associates with leaf litter, whereas feeding on termites associates with bare ground. *Hypopachus variolosus* associates with bare ground and termites, whereas *Gastrophryne elegans* associates with ants. This distinction, centered around association with bare ground, may explain why *H. variolosus* is a highly abundant winning species in disturbed habitat and *G. elegans* is a losing species. Dietary partitioning has been reported in Australian microhylids where geographically restricted species expand their diet and thus increase their chance of survival, compared to widespread species that exhibit highly specialized diets often restricted to ants (Williams et al. 2006). The third member of amphibian Guild 1, *Rhinophrynus dorsalis* has very different life history and spends most of the year buried underground and only comes to the surface to breed.

The assemblage of amphibians and reptiles present in the disturbed habitat is dominated by five ‘winning’ species: two amphibians, *Hypopachus variolosus* and *Incilius valliceps*; one snake, *Coniophanes schmidti*; and two lizards, *Corytophanes cristatus* and *Norops capito*. Although no discernable pattern could be found in the two lizard species, a pattern did emerge with the amphibians and snakes when viewed in concert. Both *H. variolosus* and *I. valliceps* associated strongly with bare ground and leaf litter in multiple habitats.

*Coniophanes schmidti* is a member of snake Guild 8 (nocturnally active frog feeding snakes) and has been observed preying upon *I. valliceps* (pers. obs.), and interestingly is the only member of the guild to show an association with bare ground. Certainly, in amphibians and snakes the association with bare ground seems to allow a species to win in disturbed habitat. The failure of the lizard models to reveal any patterns that described how natural history traits influence the ability of a species to adapt to a given habitat type is likely due to low numbers of lizard observations compared to those of amphibians and snakes. As such, further work is needed to reveal why *C. cristatus* and *N. capito* are such a major component of reptile assemblage in disturbed habitat. Additionally, the association between thermoconforming lizards and basking appears to be at first sight to misleading, however, many thermoconforming species have been observed during the study making use of singles rays of light penetrating the forest within shady areas. This association is an expression of this form of thermoregulation.

Homogenization can occur through ‘invasions’ of native species that would not normally be able to colonize forest habitats (McKinney and Lockwood 1999). For example, multiple vertebrate species that are not encountered in contiguous forest have been able to colonize remaining forest fragments from a mixed habitat matrix of forest and disturbed habitat (Gascon et al. 1999). Homogenization has been observed in amphibians in response to human-altered habitats and in most cases, generalists win at the expense of losing specialist species (Vallan et al. 2004; Hirshfeld and Rödel 2017). Metadata studies into patterns of global homogenization in amphibians identified that species/clades that are often most at risk are those with direct-developing tadpoles, for example, salamanders of the genus *Bolitoglossa*, and frogs of the genera *Pristimantis* and *Craugastor* (Nowakowski et al. 2018). Species that tend to do well due to homogenization are those that reproduce in standing pools of water (often associated with livestock water holes in an agricultural landscape), such as certain hylid frog groups.

This study identified that an association with bare ground, and therefore a tolerance of drier habitats enables a small number of amphibian and snake species to utilize the disturbed forest close to agricultural land in PNLT. This is broadly consistent with other studies that have identified a reduced diversity of microhabitats, in particular reduction of leaf litter, bromeliads, and water bodies, influence tropical amphibian and reptile assemblage structure (Vallan et al. 2004; Gallmetzer and Schulze 2015; Hernandez-Ordoñez et al. 2015; Hirschfeld and Rödel 2017; Nowakowski et al. 2018). Those species identified in this study as winning species occurred in all three habitat types, suggesting an ability to adapt to novel or disturbed habitats. Of major conservation concern, this suggests that continued forest fragmentation in PNLT and the wider Mayan Biosphere Reserve will result in increased edge effects, a greater proportion of remaining forest kept in an early successional state, and with a highly reduced, and homogenized, amphibian and reptile assemblage of Northern Guatemala. In this case, we can predict that species such *Incilius valliceps*, *Hypopachus* variolosus, and *Coniophanes schmidti* will be the winning species that replace the losing species in the assemblage of the region. The homogenization of species assemblages is not restricted to amphibians and reptiles and has been reported across a wide variety of taxa (McKinney and Lockwood 1999; Newbold et al. 2018). With rates of deforestation and fragmentation increasing across the tropics, there is a global threat of homogenization where generalist species win in favour of ‘losing’ specialist species (Tabarelli et al. 2012; Dang et al. 2019; Vargas Zeppetello et al. 2020). As such there is an urgent need to reduce the rate of habitat loss and fragmentation in the tropics to halt the continued homogenization of tropical faunal assemblages.

The WLR concept is a theoretical framework for species responses to habitat degradation, our study serves as an initial baseline to add empirical evidence to this concept in terms of how amphibians and reptiles respond to these anthropogenic influences. To increase future accuracy, further studies should aim to decipher the differences between obvious positive and negative traits with those that might be more generalist in ecology. Our study serves as a model for how to integrate WLR concept into long term monitoring programs for any species community. Such application has an immediate usefulness for the prediction of population increases or declines across taxa in the tropics.

## Supplementary Information

Below is the link to the electronic supplementary material.

Supplementary file1 (DOCX 9894 KB)

## Data Availability

Data + R code for this project is available at: 10.5281/zenodo.14775627.
