# Supplementary material for: Natural history traits influence winners and losers for herpetological communities in disturbed tropical habitats
Source: Oecologia. 2025 Mar 12;207(3):52. doi: 10.1007/s00442-025-05691-7 (PMC11897091; doi:10.1007/s00442-025-05691-7)

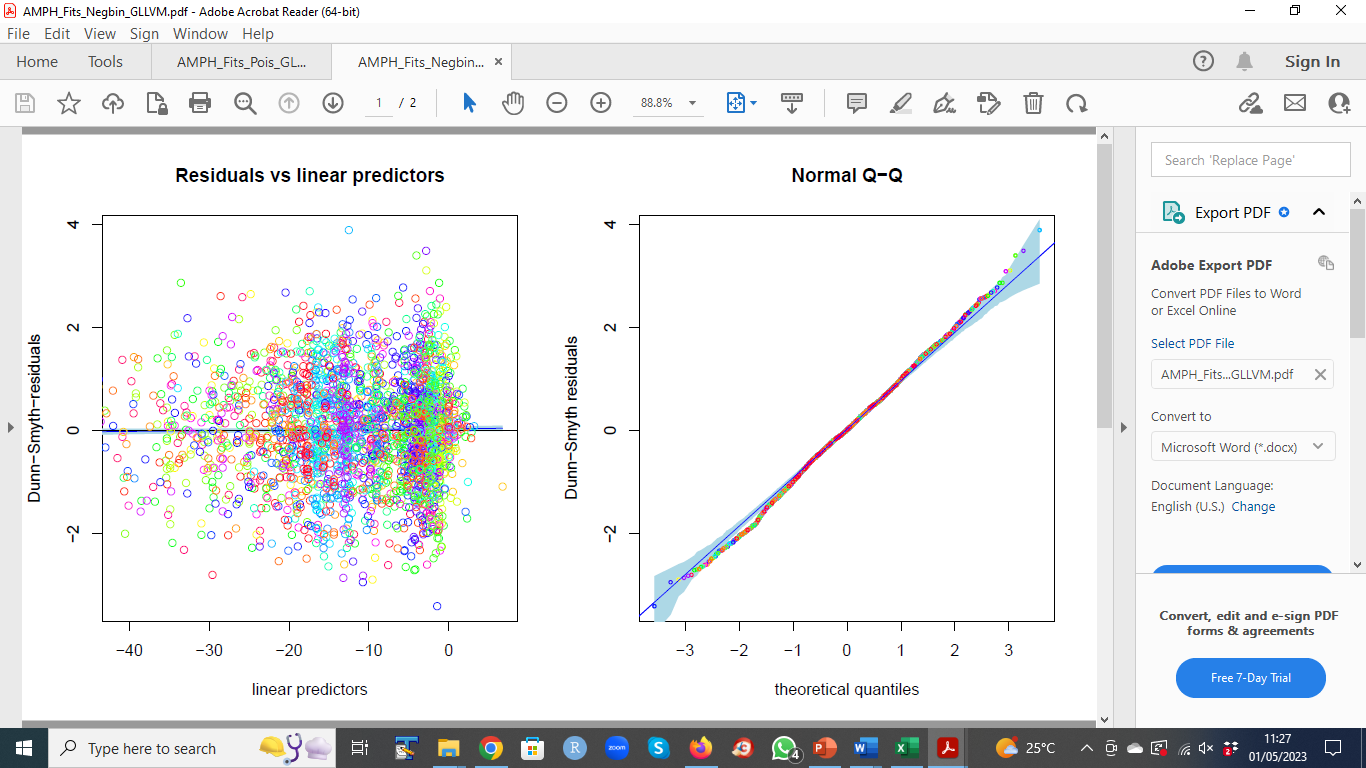

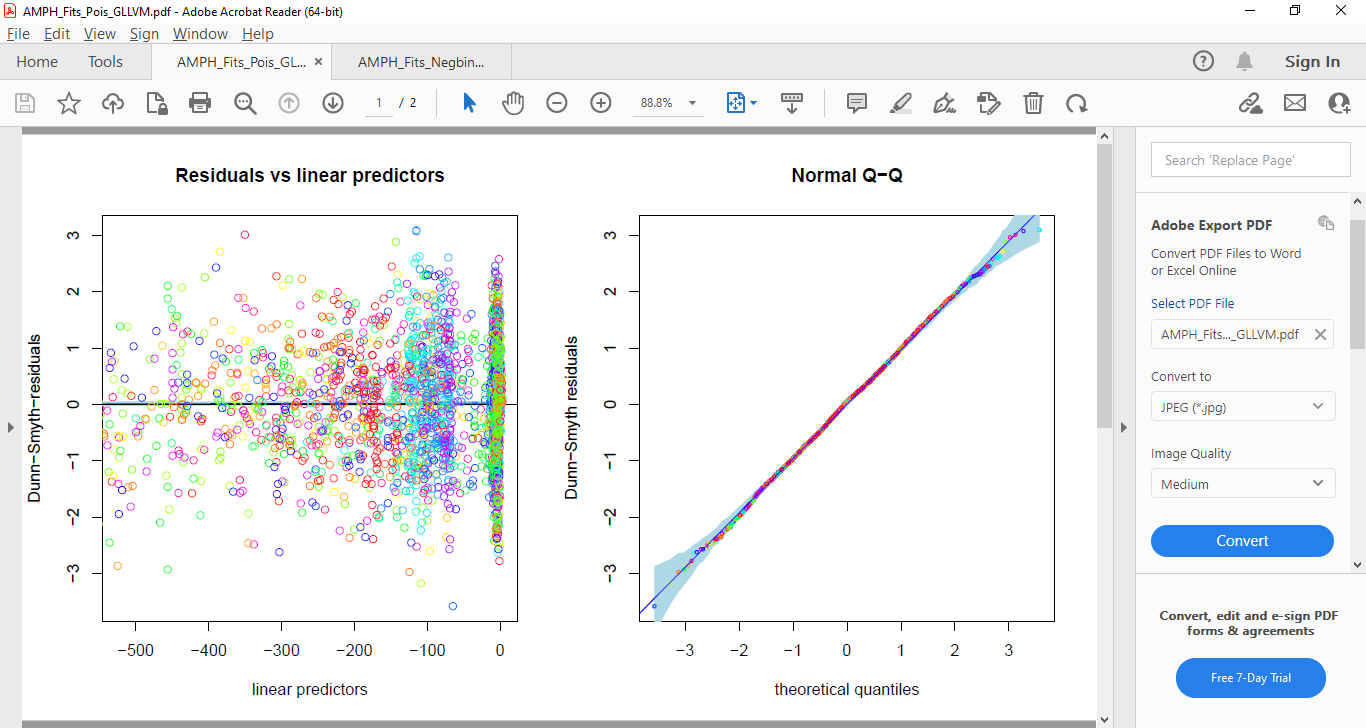
Supplementary Material

**Figure S.1:** Dunn-Smyth residuals and Q-Q plots for the amphibian GLLVM showing model fits for Poisson distribution (above) and negative binomial distribution (below).


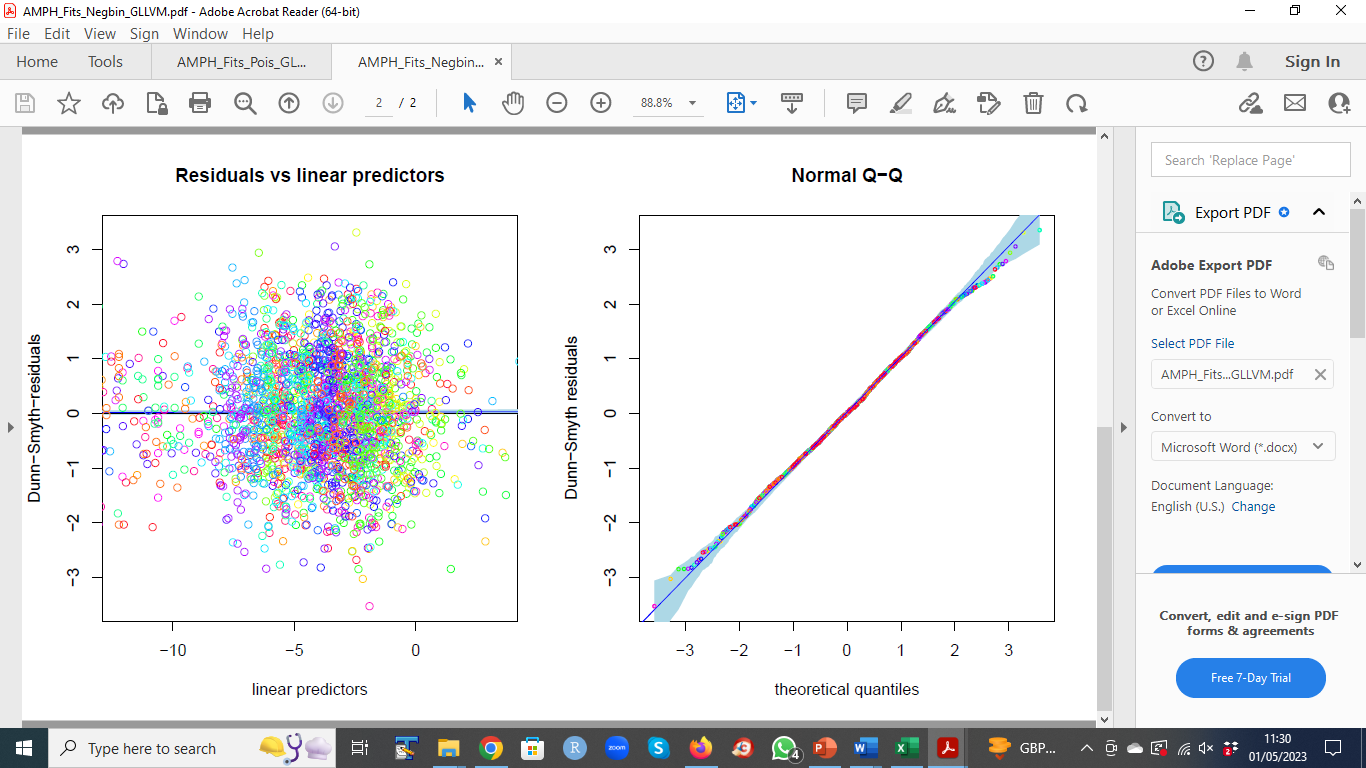

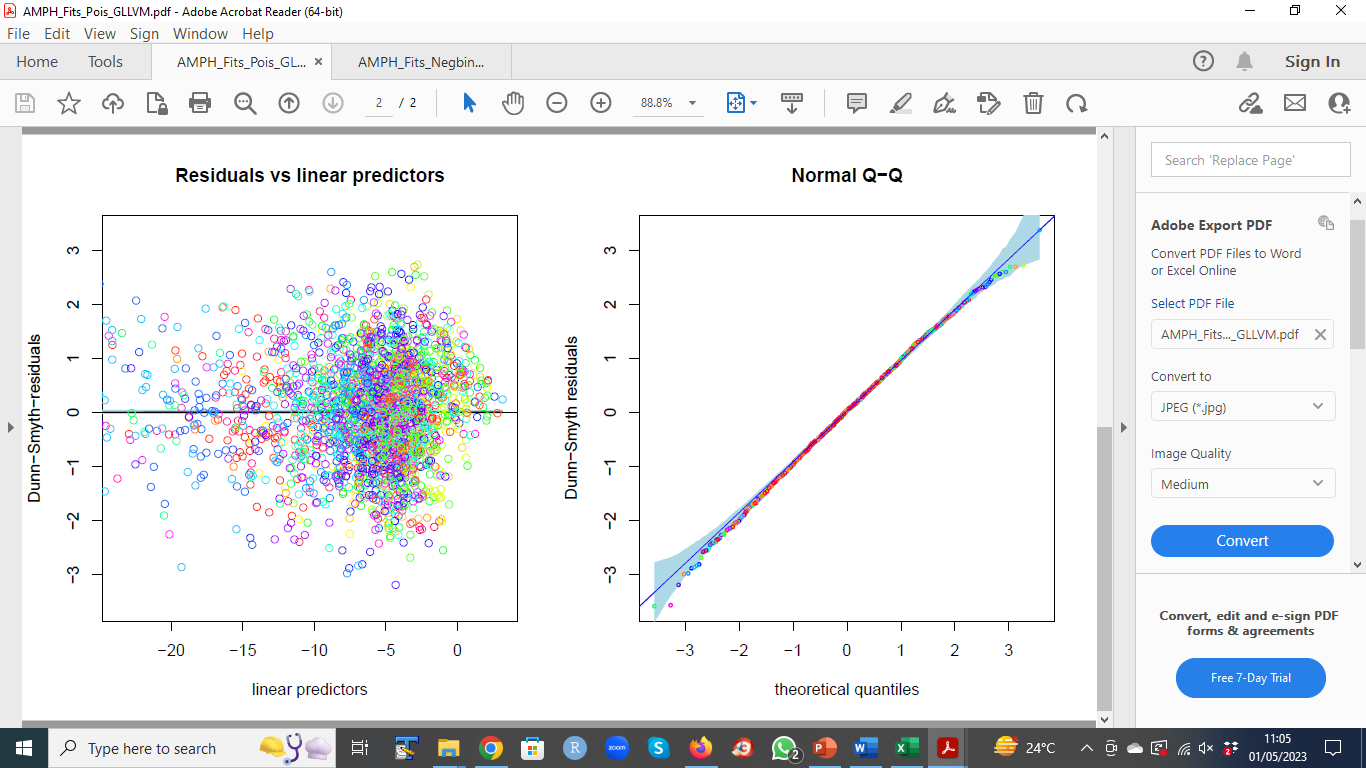
**Figure S.2:** Dunn-Smyth residuals and Q-Q plots for the amphibian LV Trait GLM showing model fits for Poisson distribution (above) and negative binomial distribution (below).


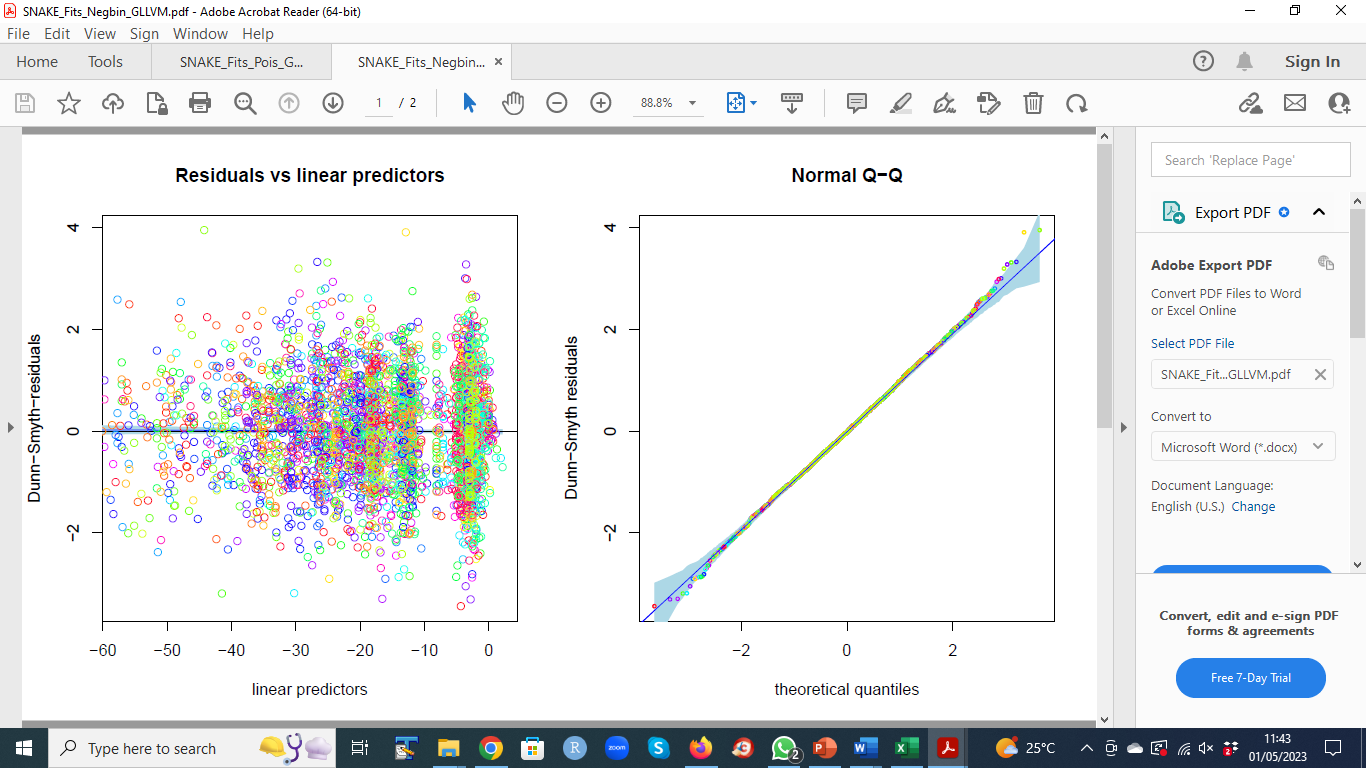

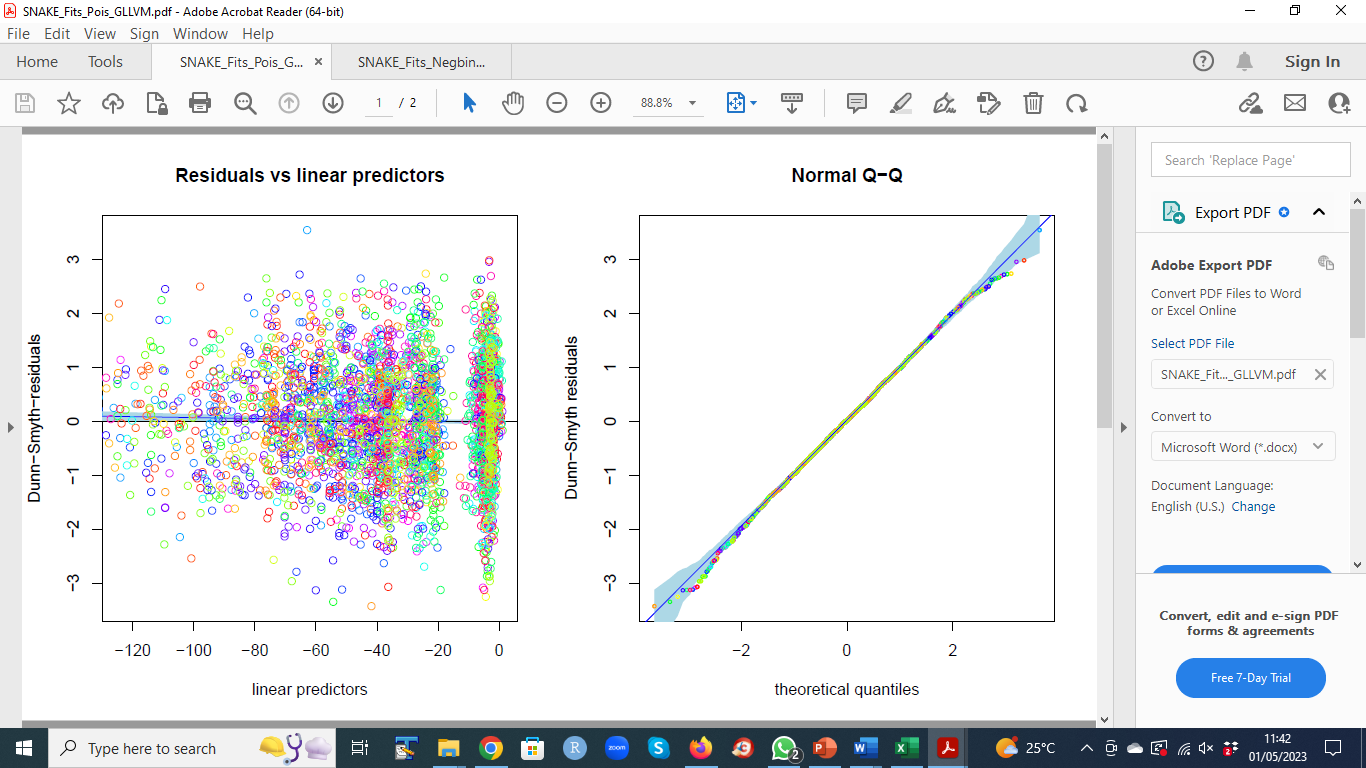


**Figure S.3:** Dunn-Smyth residuals and Q-Q plots for the snake GLLVM showing model fits for Poisson distribution (above) and negative binomial distribution (below).


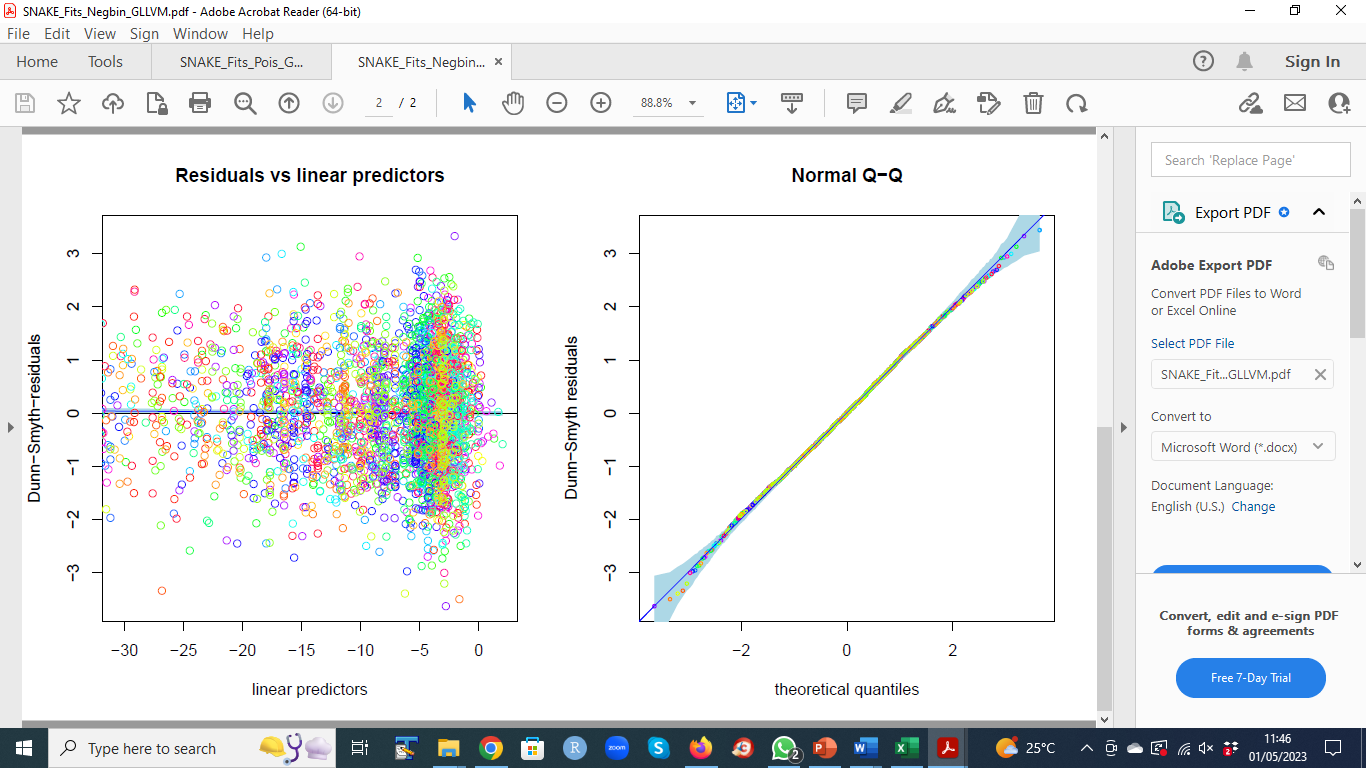

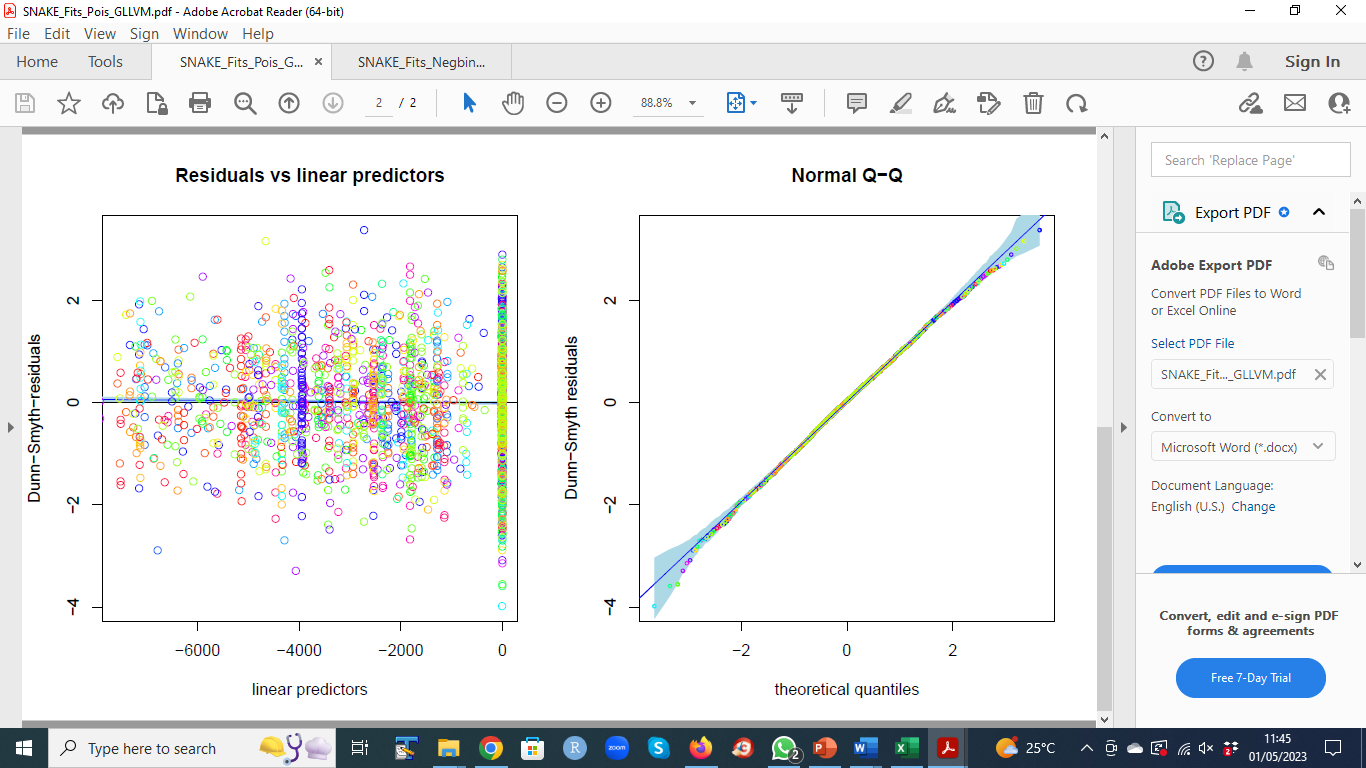


**Figure S.4:** Dunn-Smyth residuals and Q-Q plots for the snake LV Trait GLM showing model fits for Poisson distribution (above) and negative binomial distribution (below).


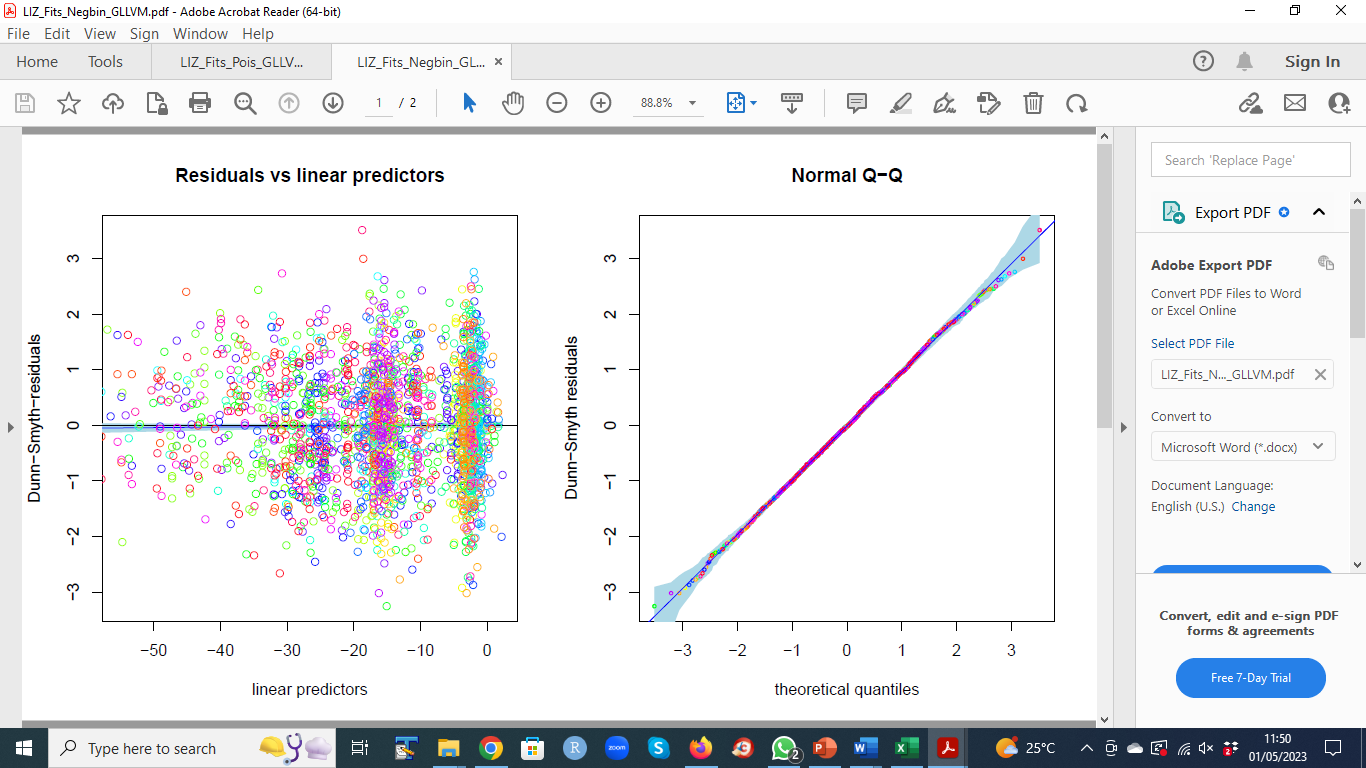

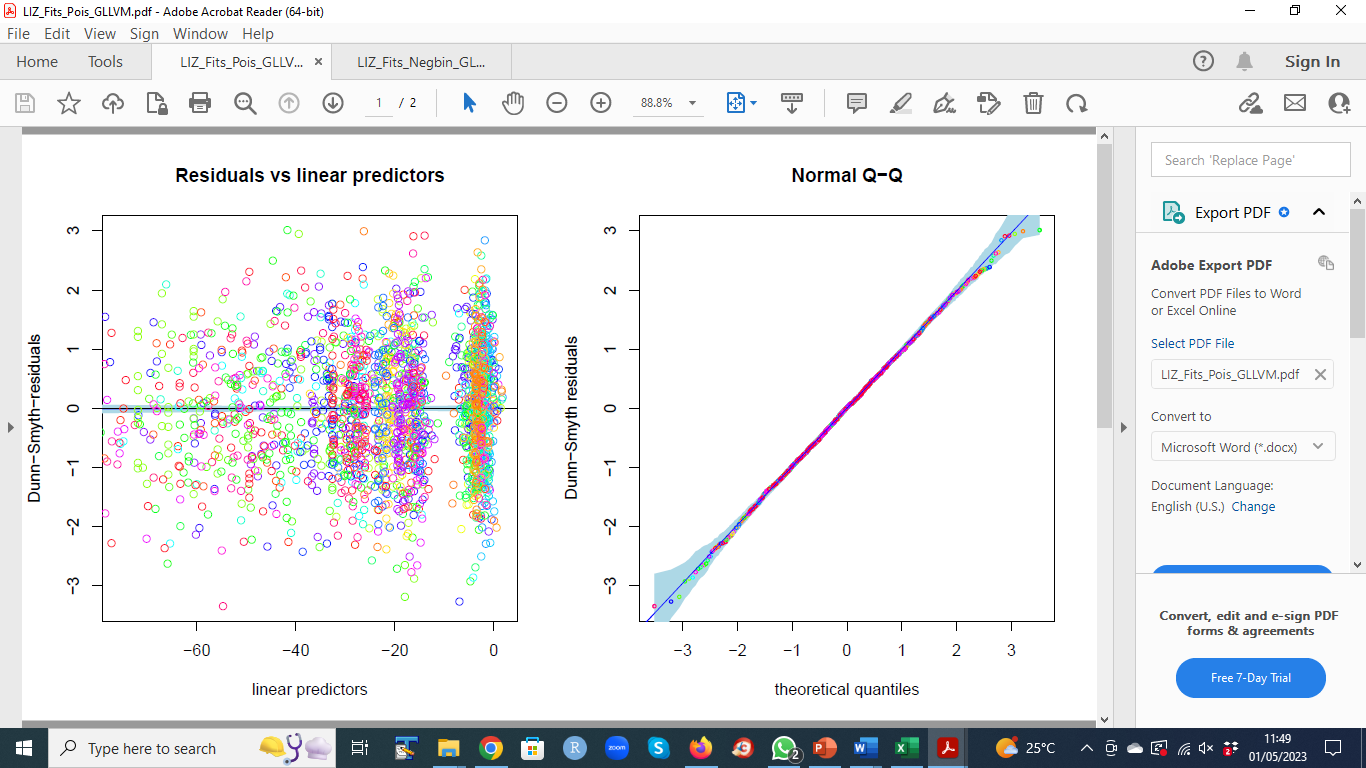


**Figure S.5:** Dunn-Smyth residuals and Q-Q plots for the lizard GLLVM showing model fits for Poisson distribution (above) and negative binomial distribution (below).


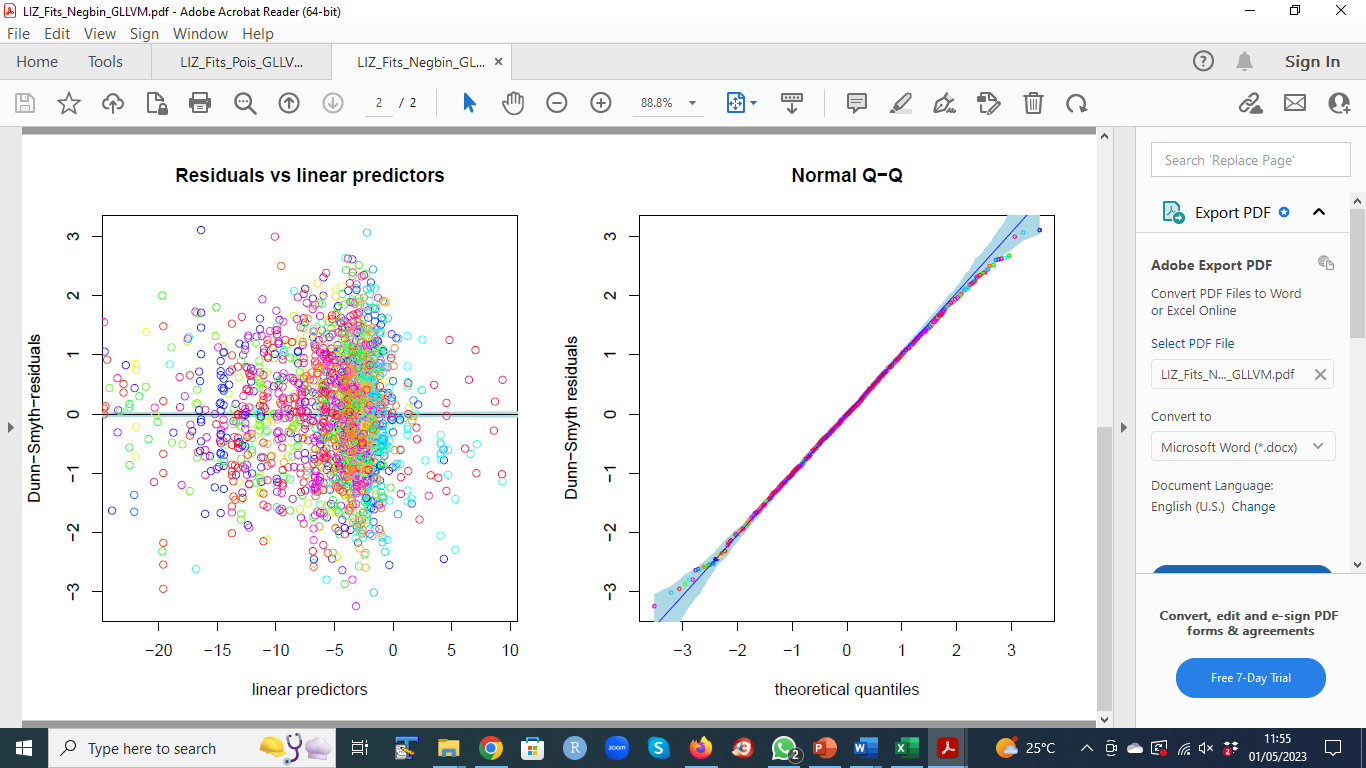

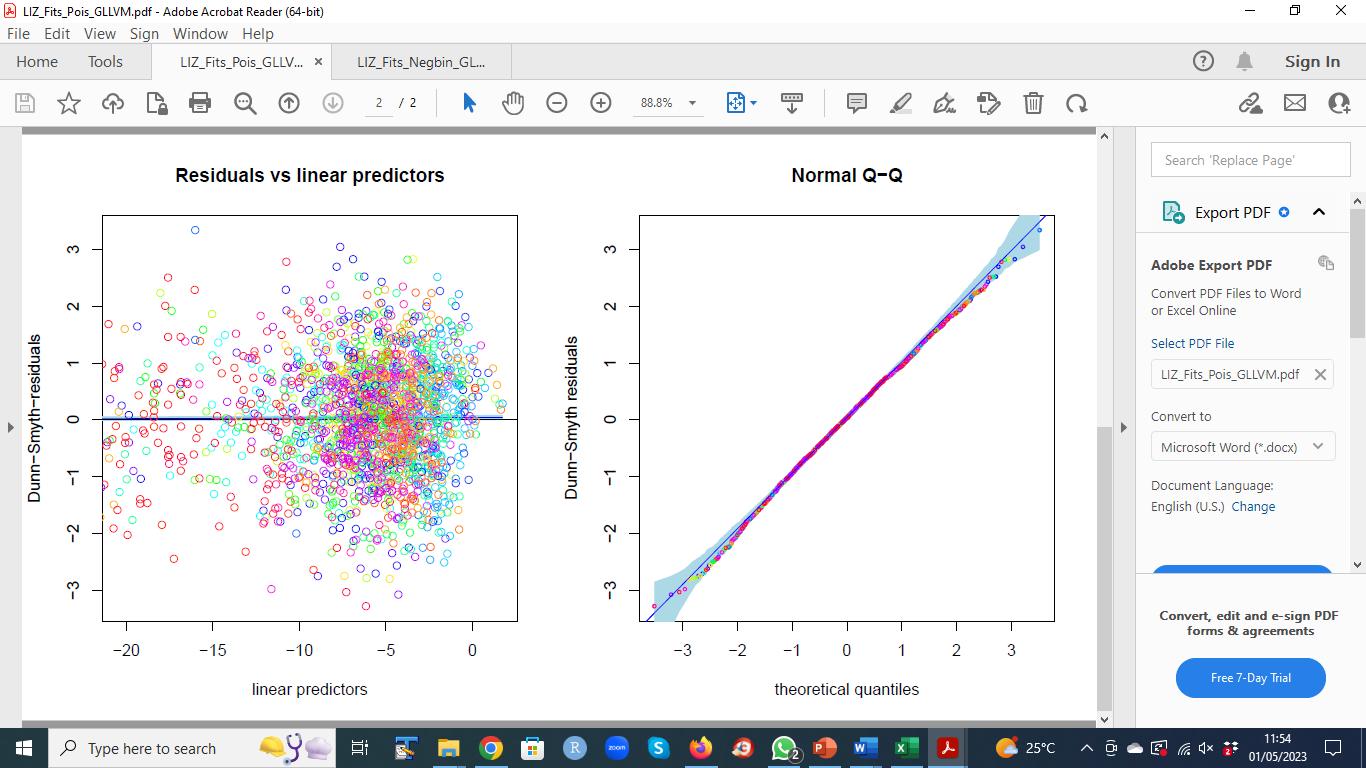
**Figure S.6:** Dunn-Smyth residuals and Q-Q plots for the lizard LV Trait GLM showing model fits for Poisson distribution (above) and negative binomial distribution (below).


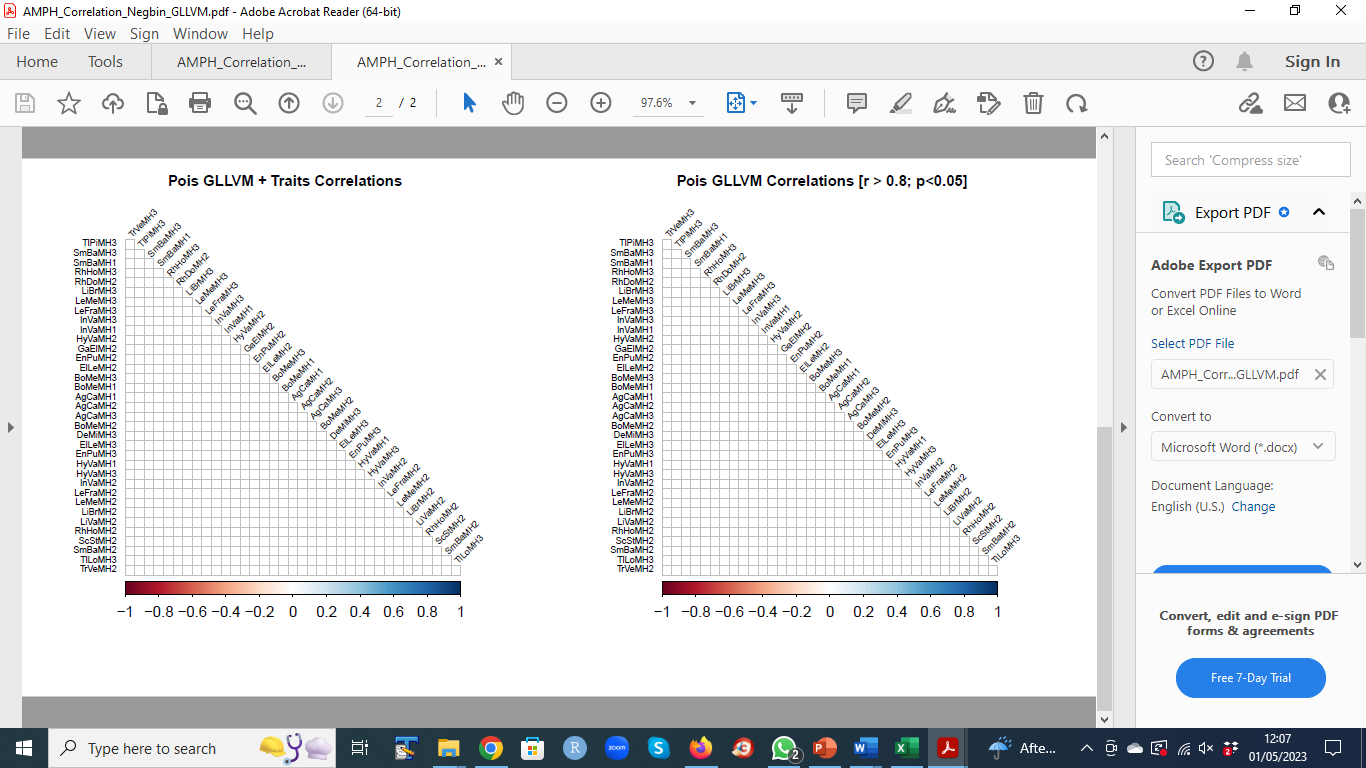

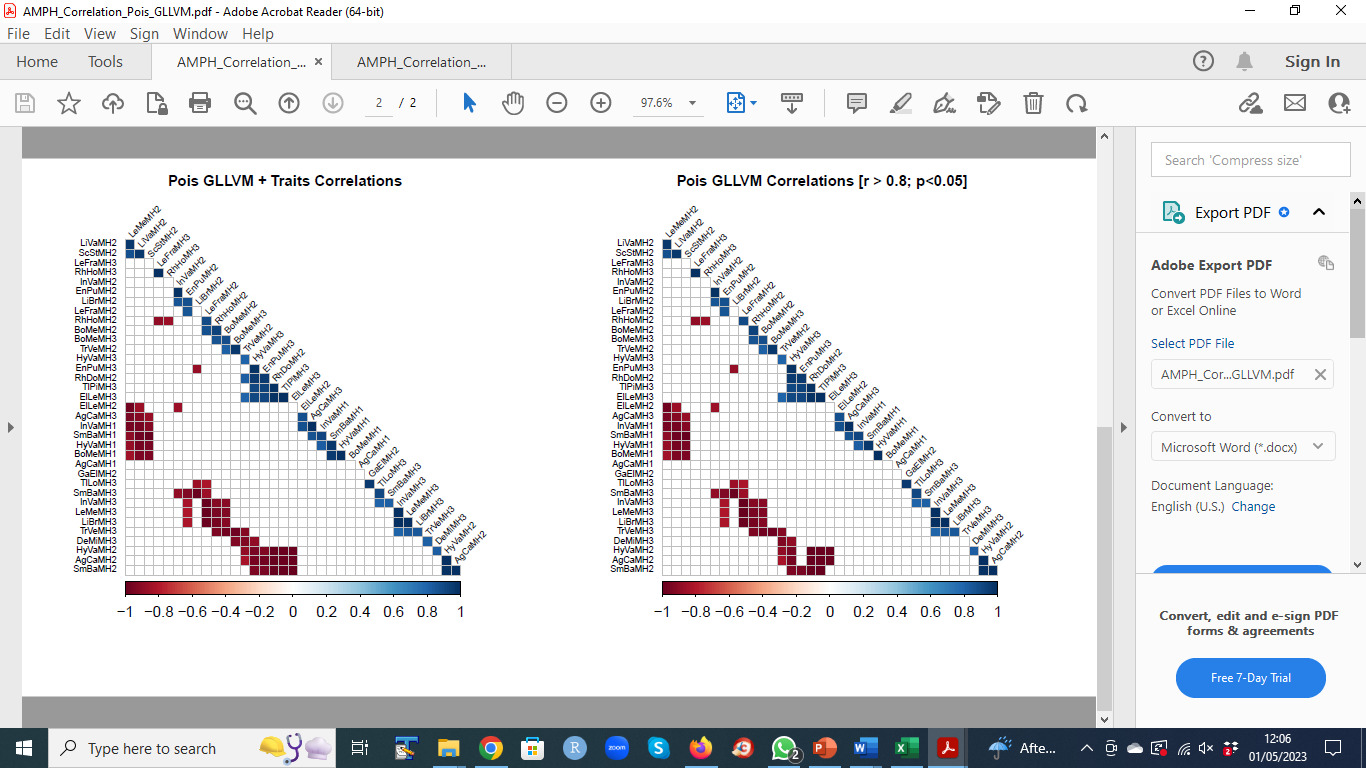

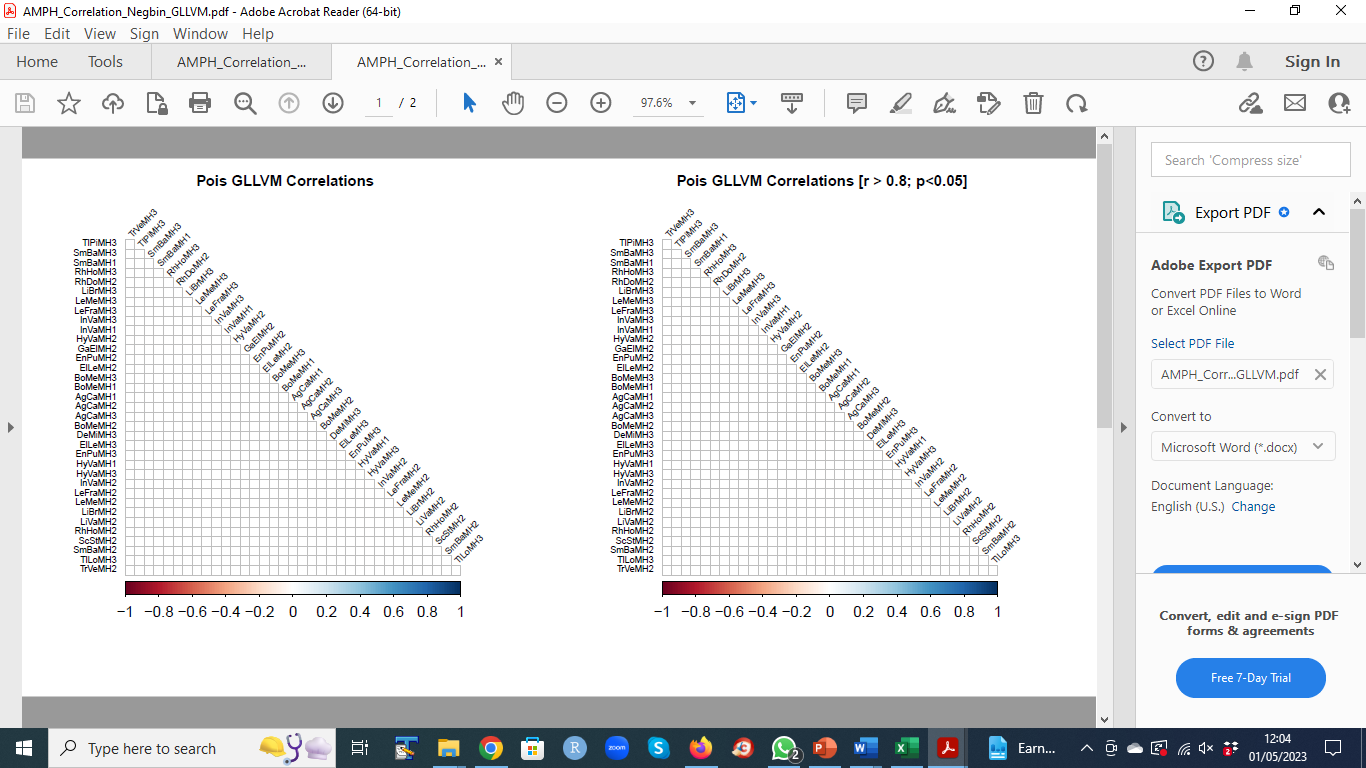

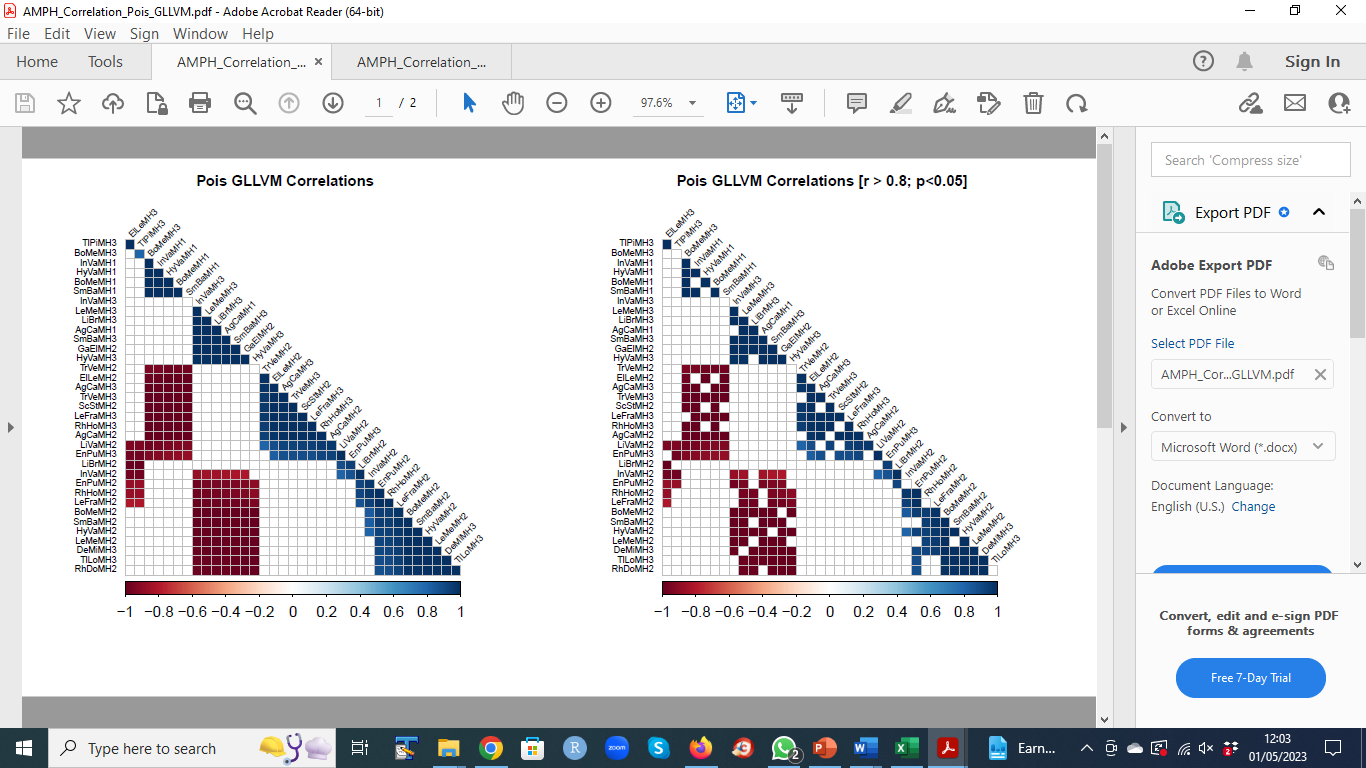


**Figure S.7:** Comparison of Poisson distribution (left) and negative binomial distribution (right) correlation plots for the amphibian GLLVM (above) and LV Trait GLM (below). Red squares show negative correlation, blue squares show positive correlation. The strength of the colour indicates strength of the correlation.


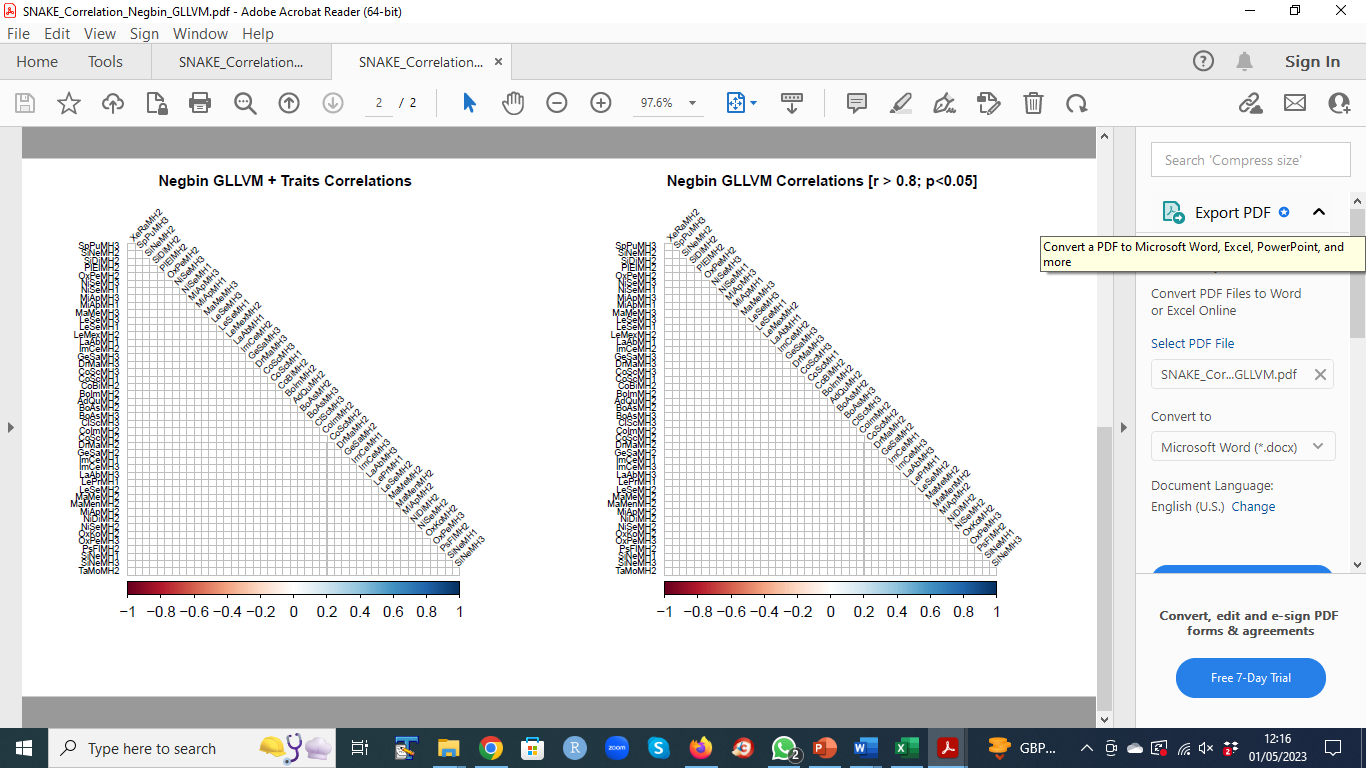

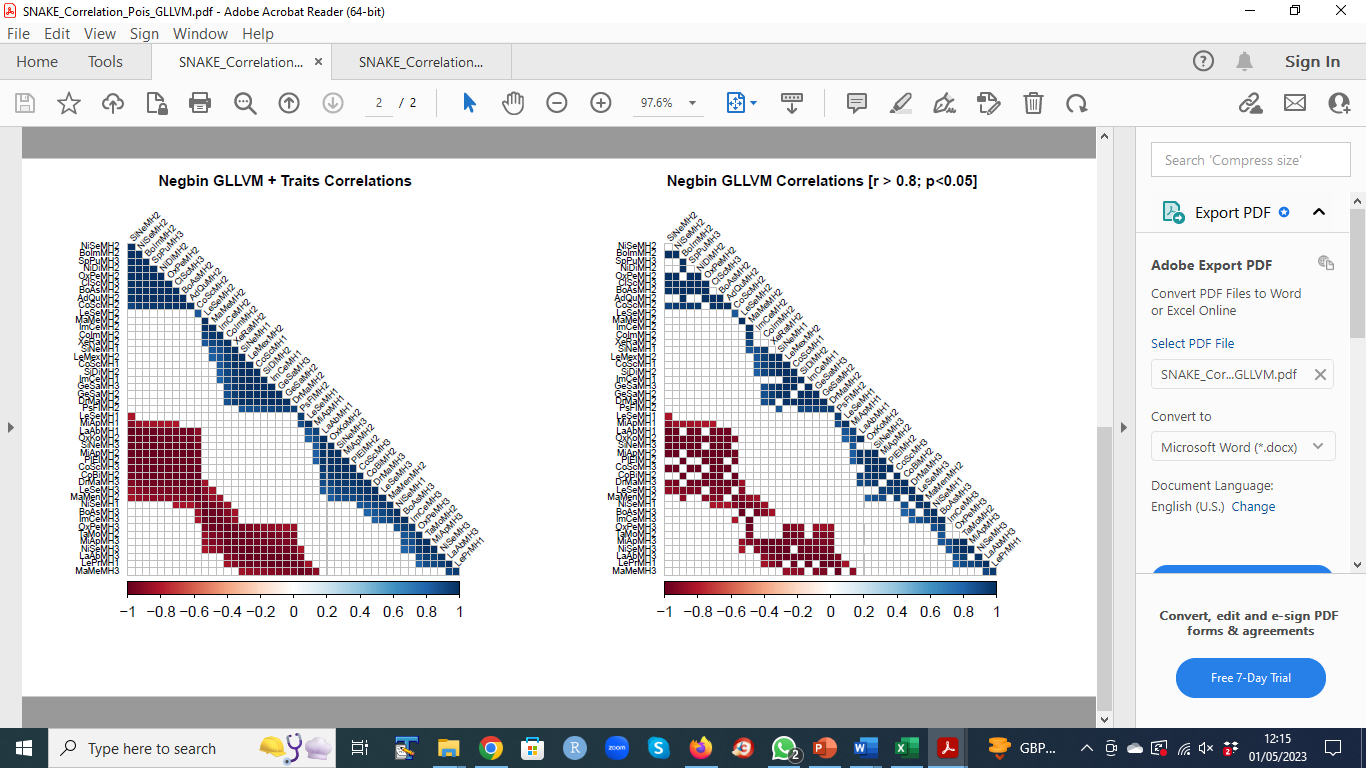

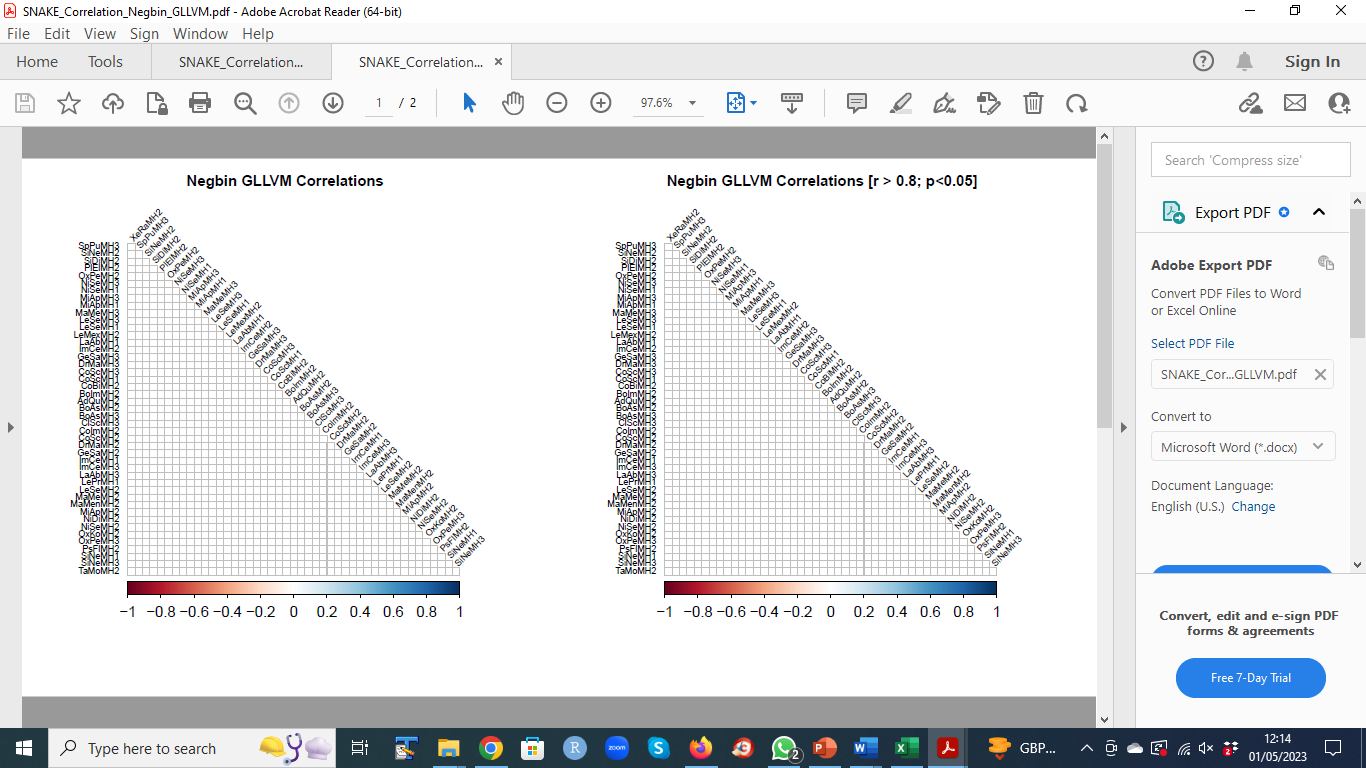

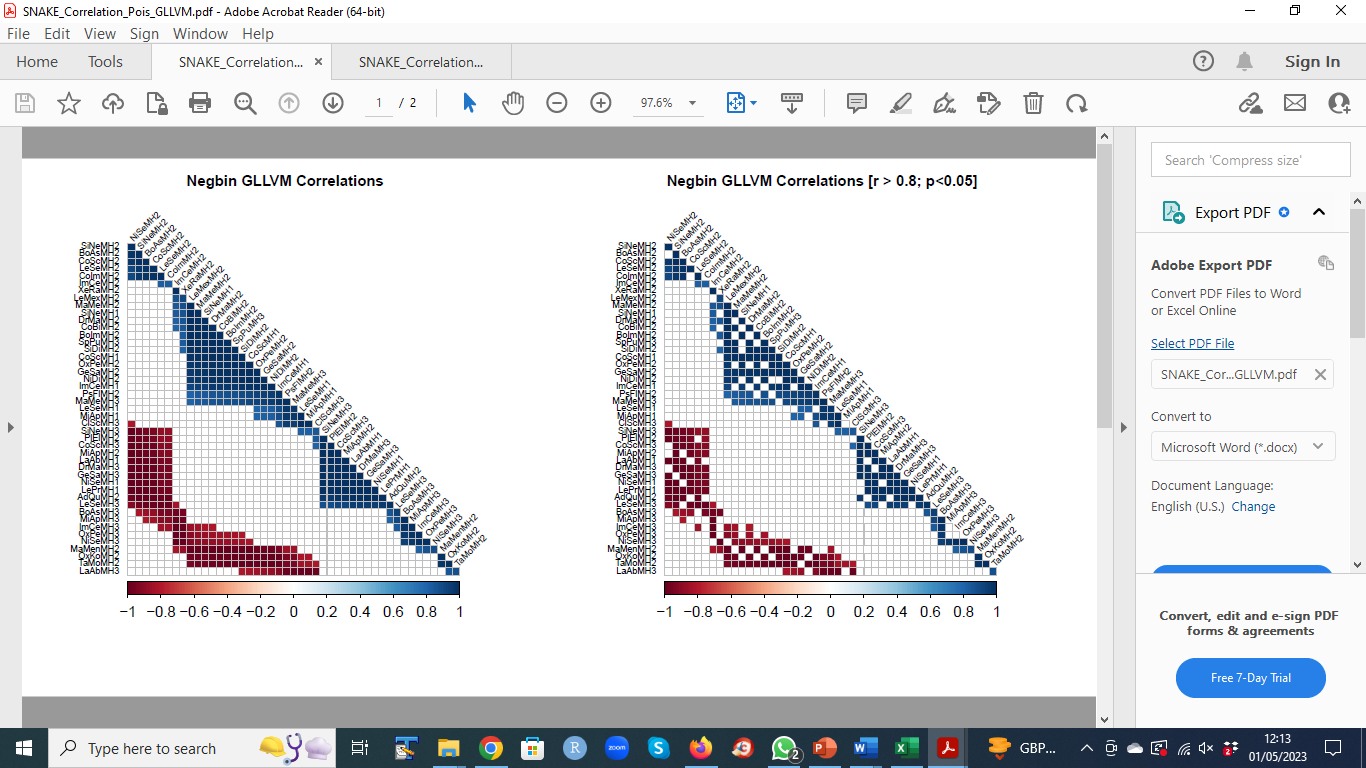
**Figure S.8:** Comparison of Poisson distribution (left) and negative binomial distribution (right) correlation plots for the snake GLLVM (above) and LV Trait GLM (below). Red squares show negative correlation, blue squares show positive correlation. The strength of the colour indicates strength of the correlation.


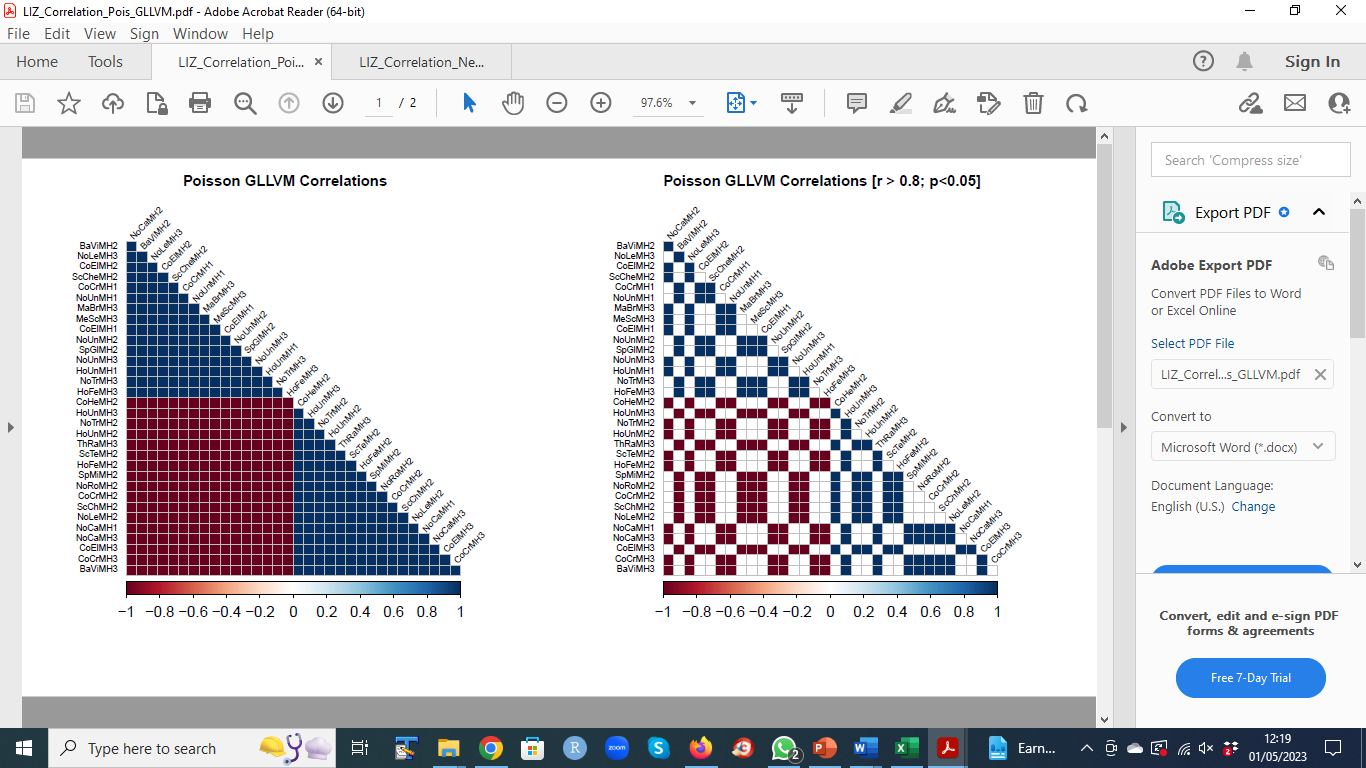

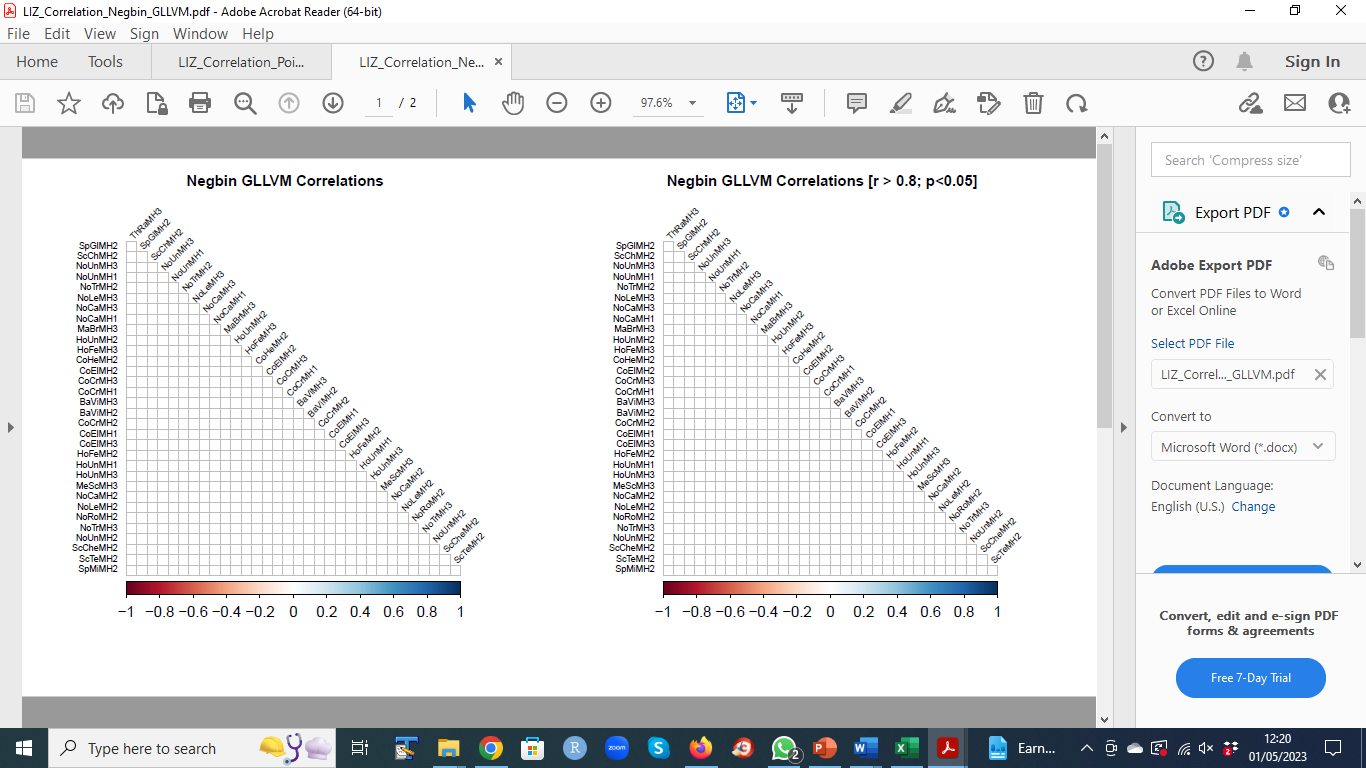

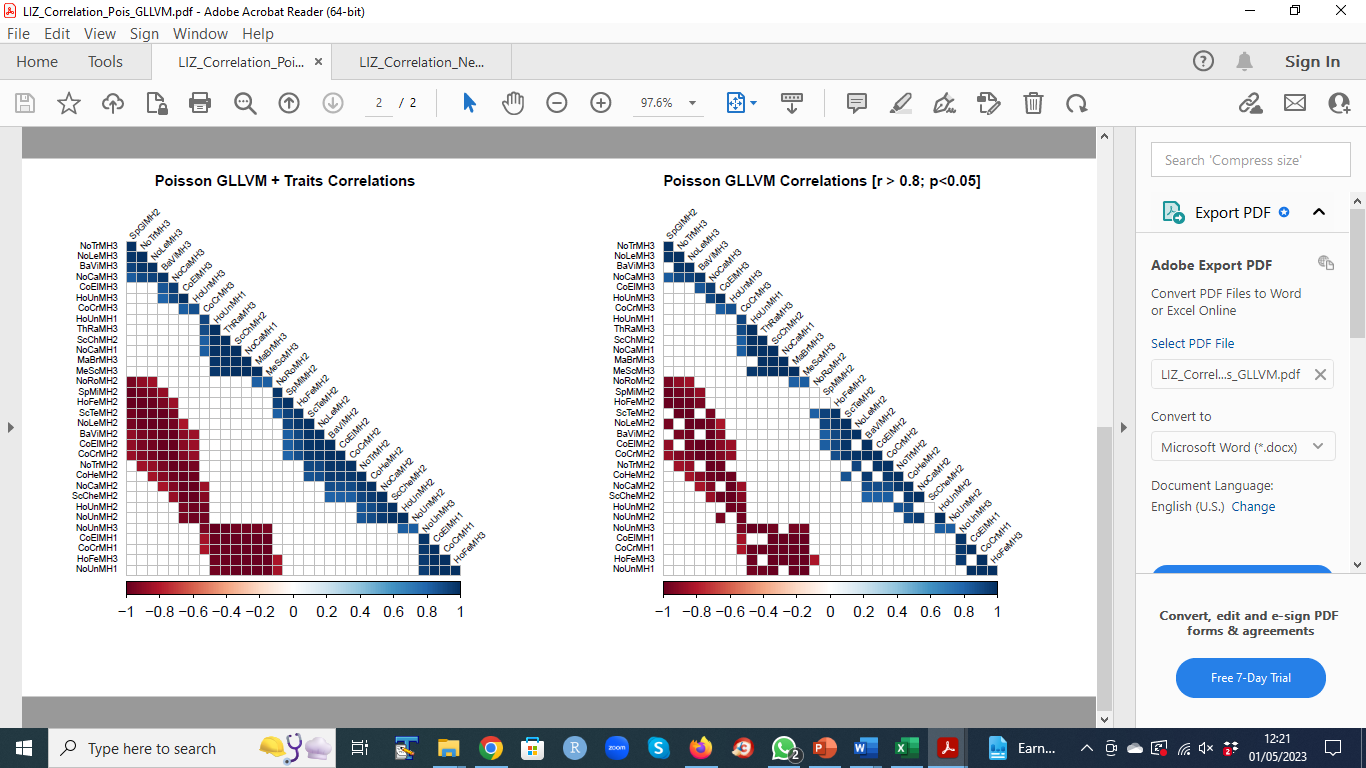

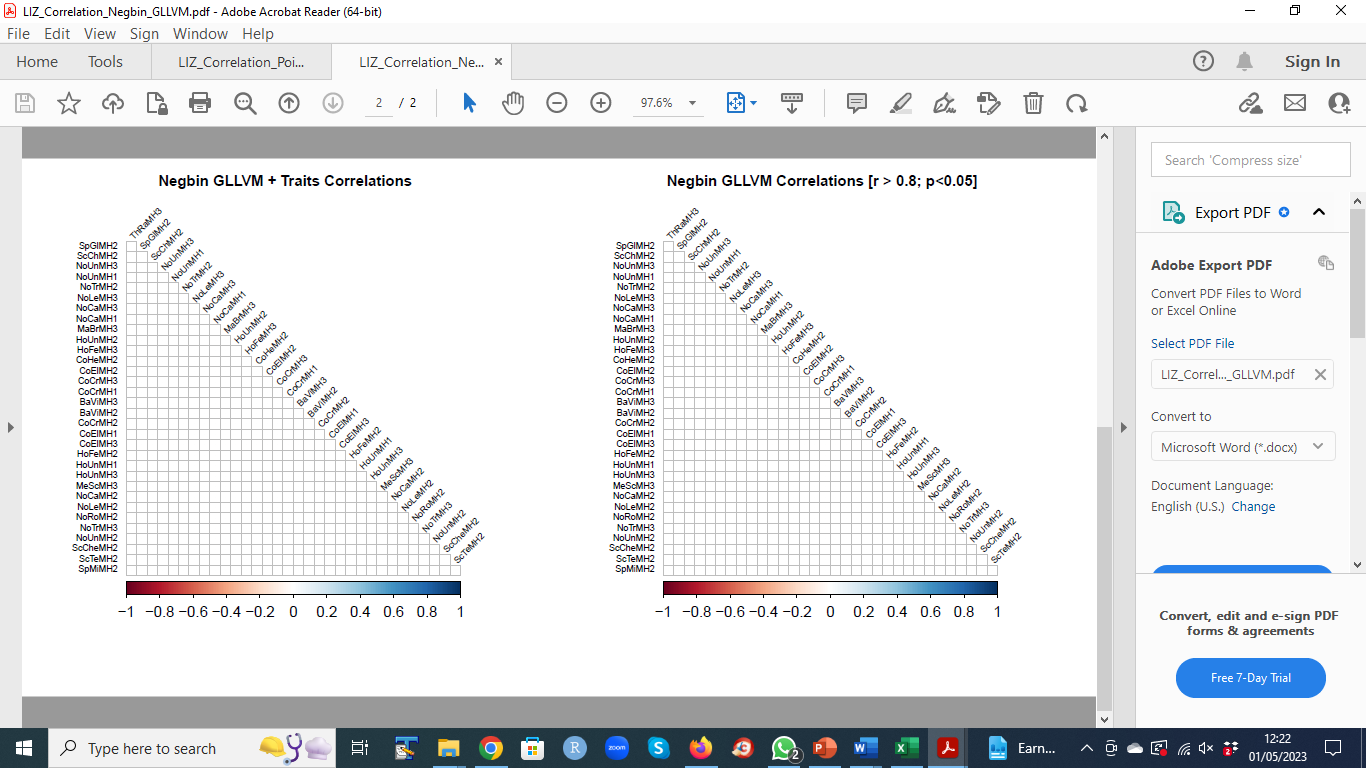
**Figure S.9:** Comparison of Poisson distribution (left) and negative binomial distribution (right) correlation plots for the lizard GLLVM (above) and LV Trait GLM (below). Red squares show negative correlation, blue squares show positive correlation. The strength of the colour indicates strength of the correlation.


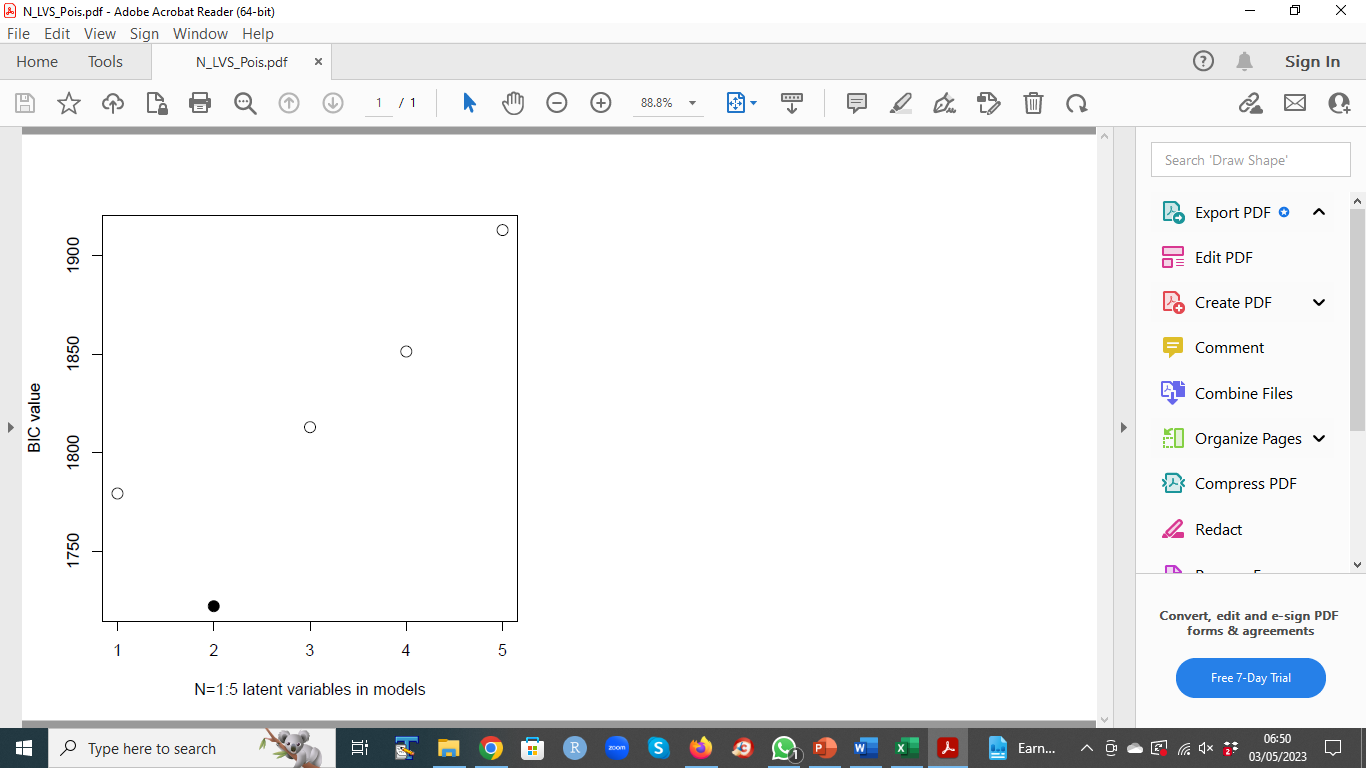

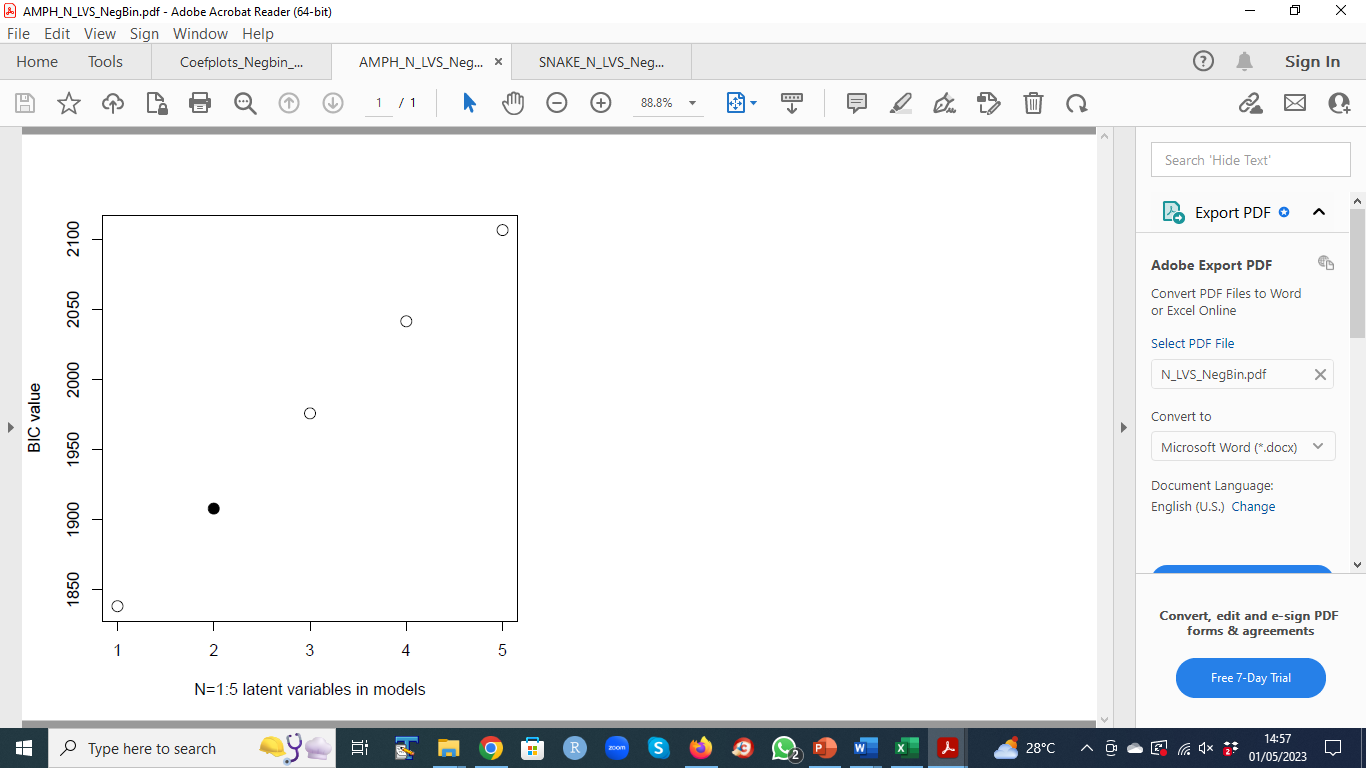


**Figure S.10:** BIC scores for the selection of number of latent variables for the amphibian GLLVM, showing Poisson distributed model (left) and negative binomial model (right).


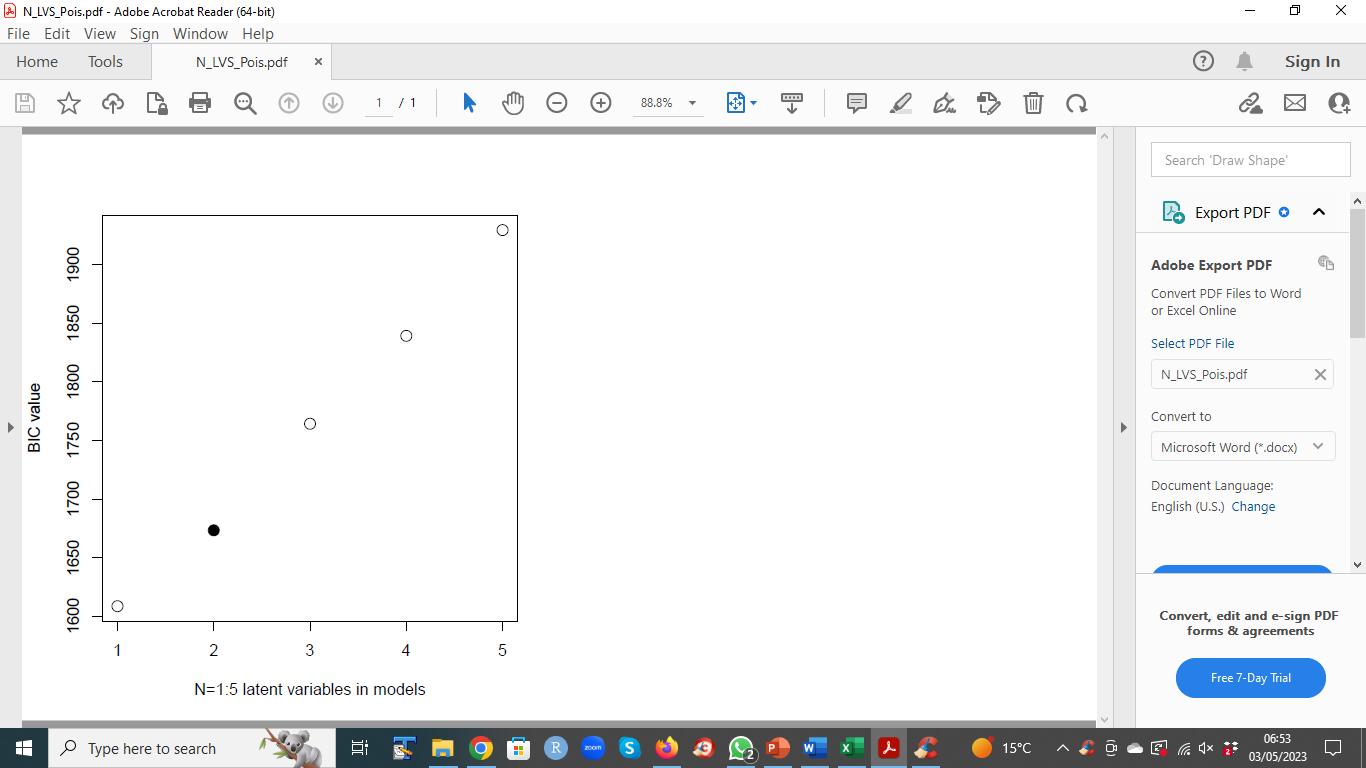


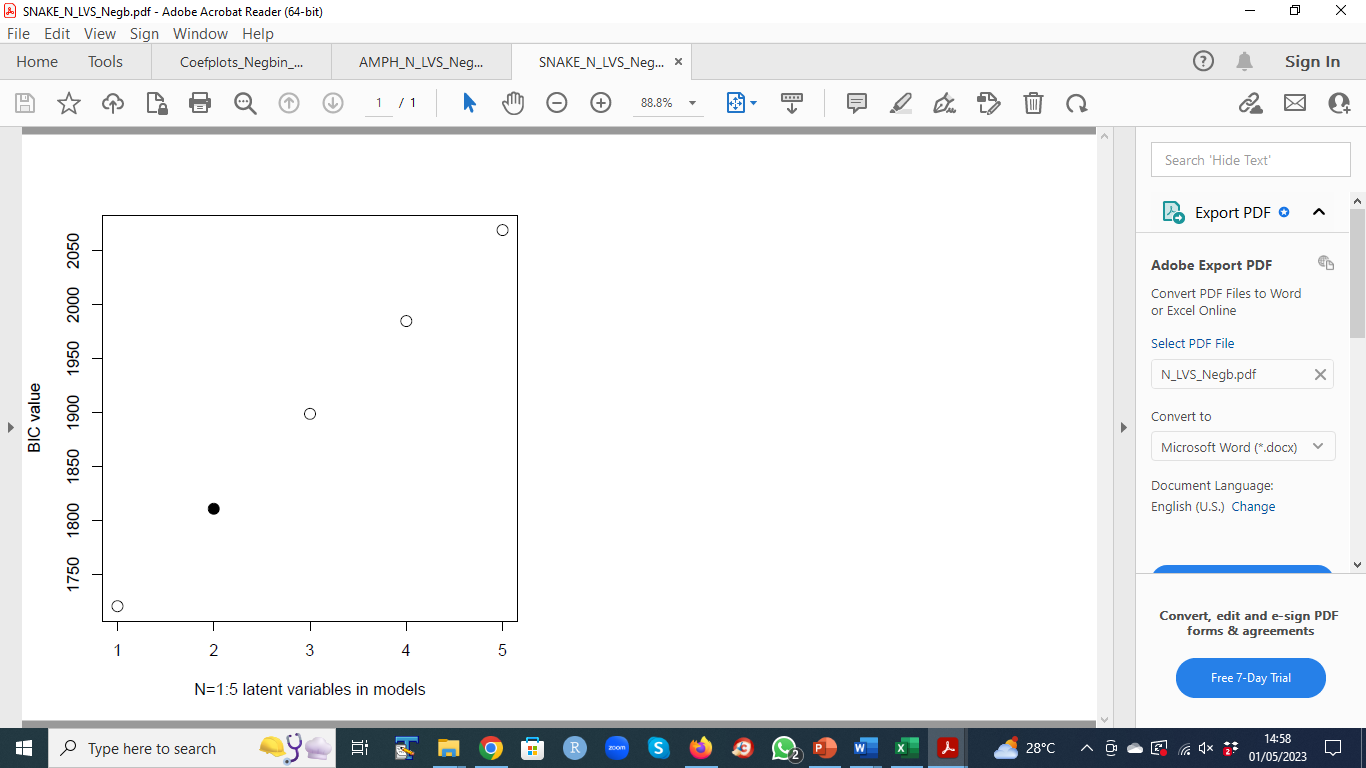
**Figure S.11:** BIC scores for the selection of number of latent variables for the snake GLLVM showing Poisson distributed model (left) and negative binomial model (right).


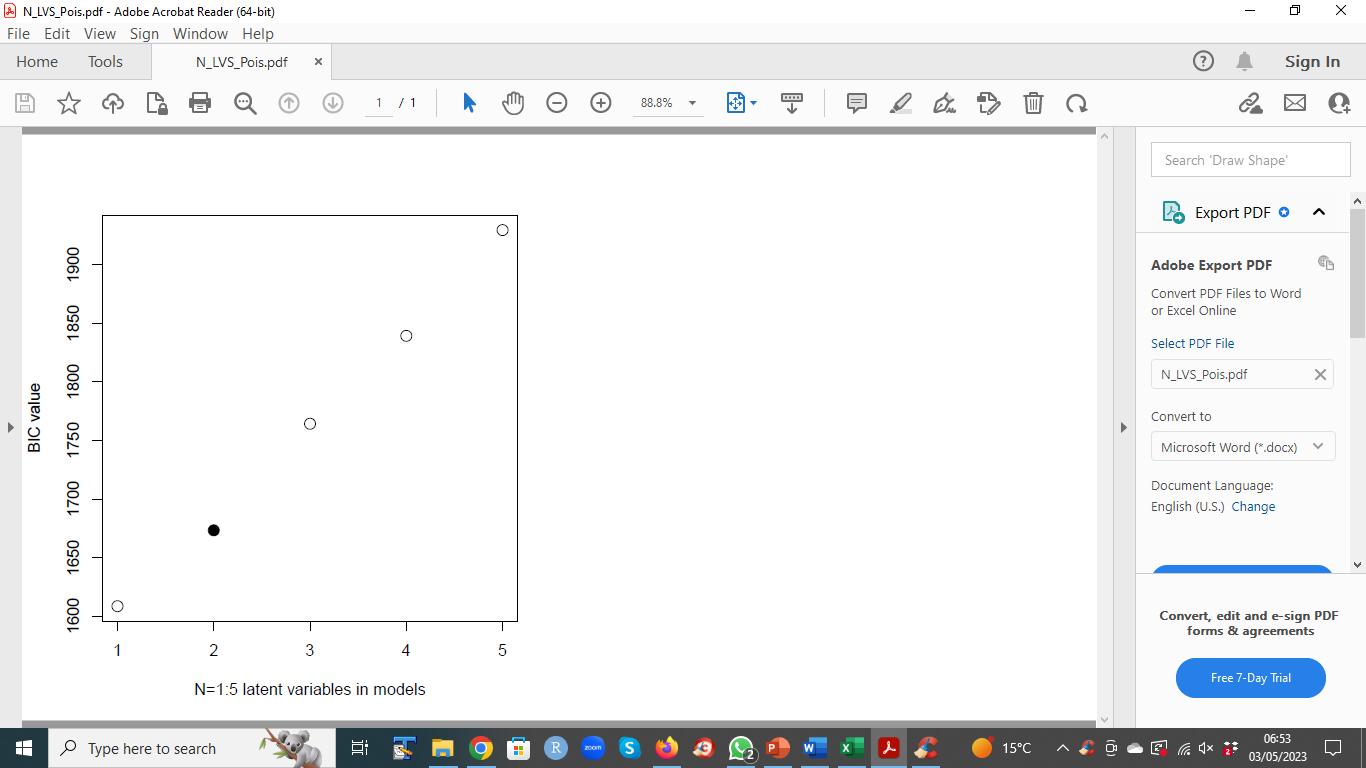

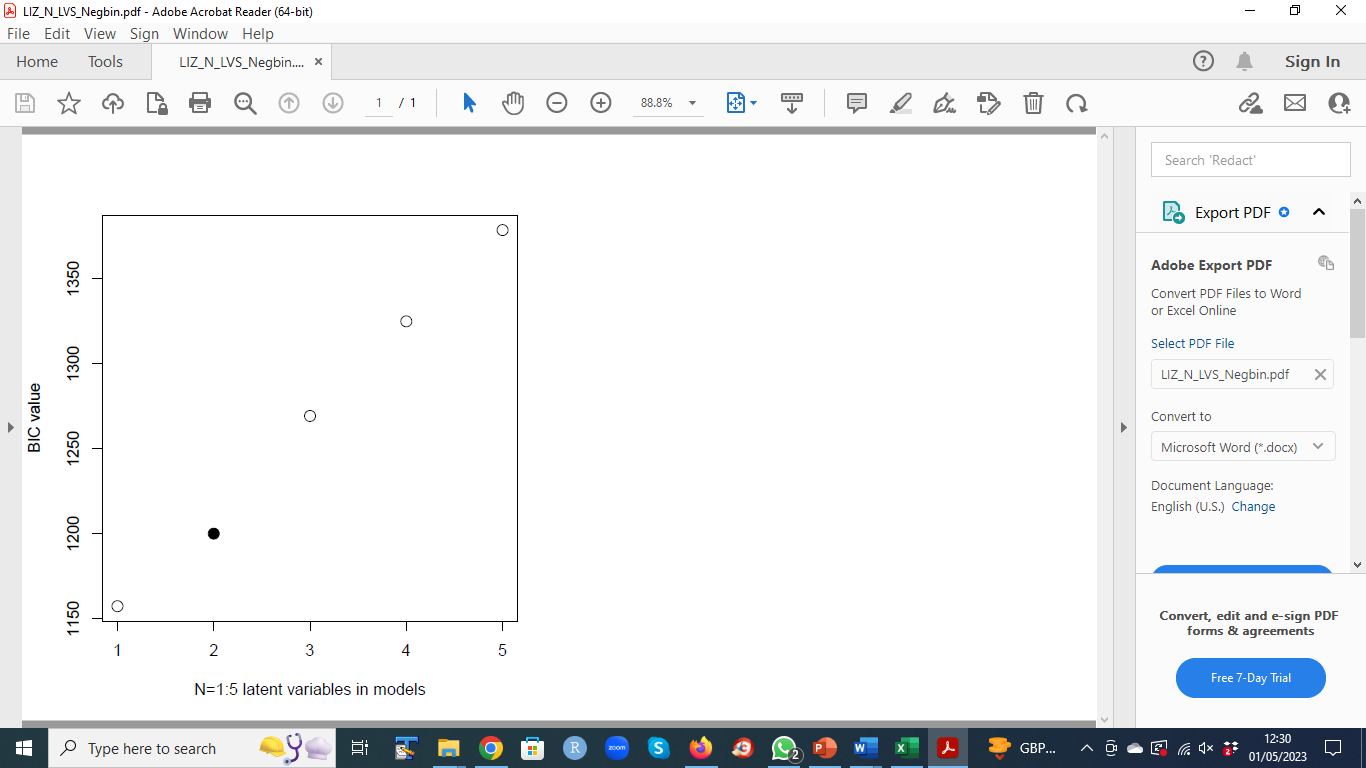
**Figure S.12:** BIC scores for the selection of number of latent variables for the lizard GLLVM showing Poisson distributed model (left) and negative binomial model (right).


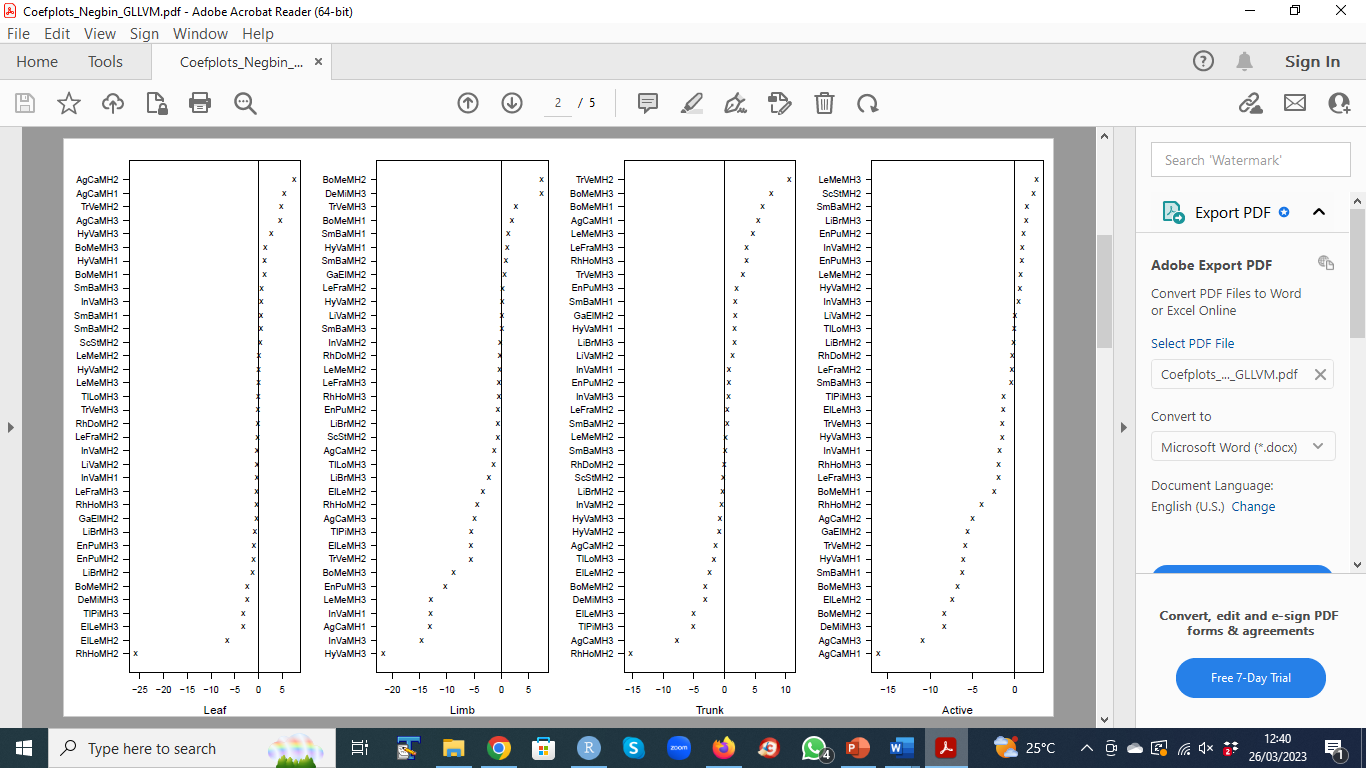

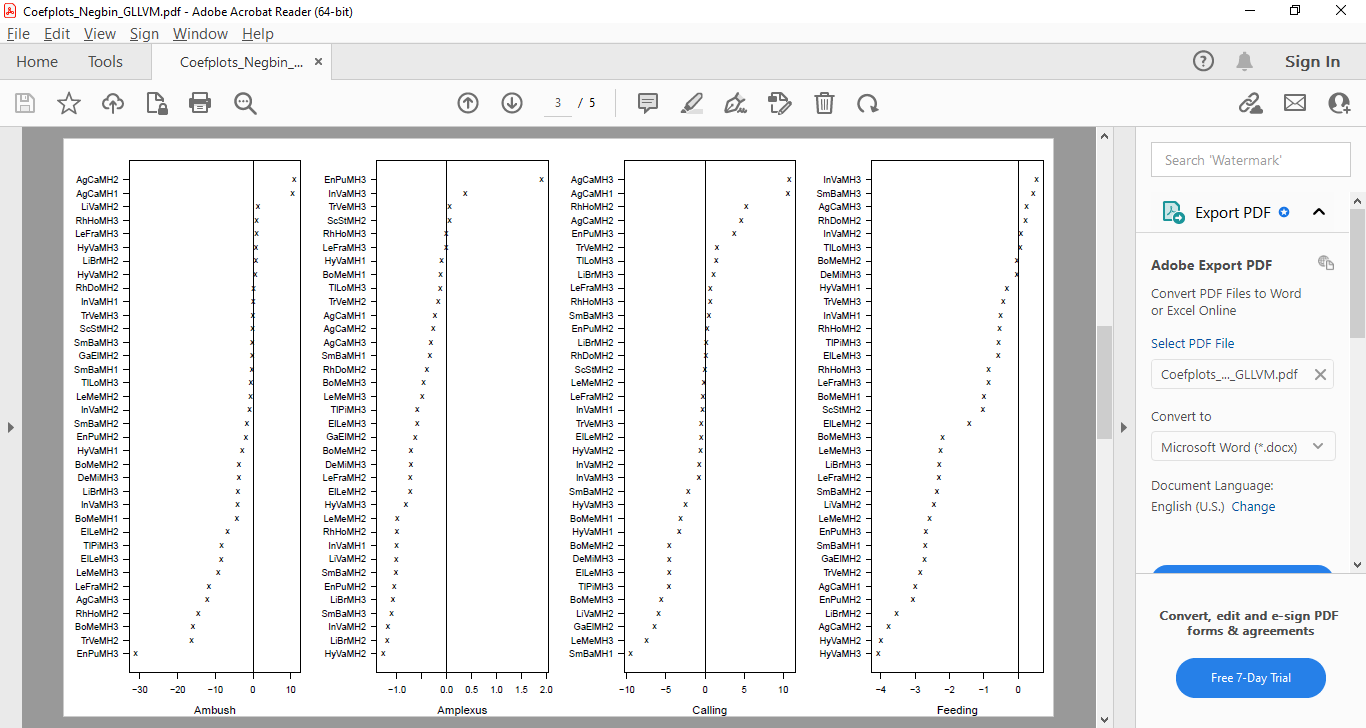

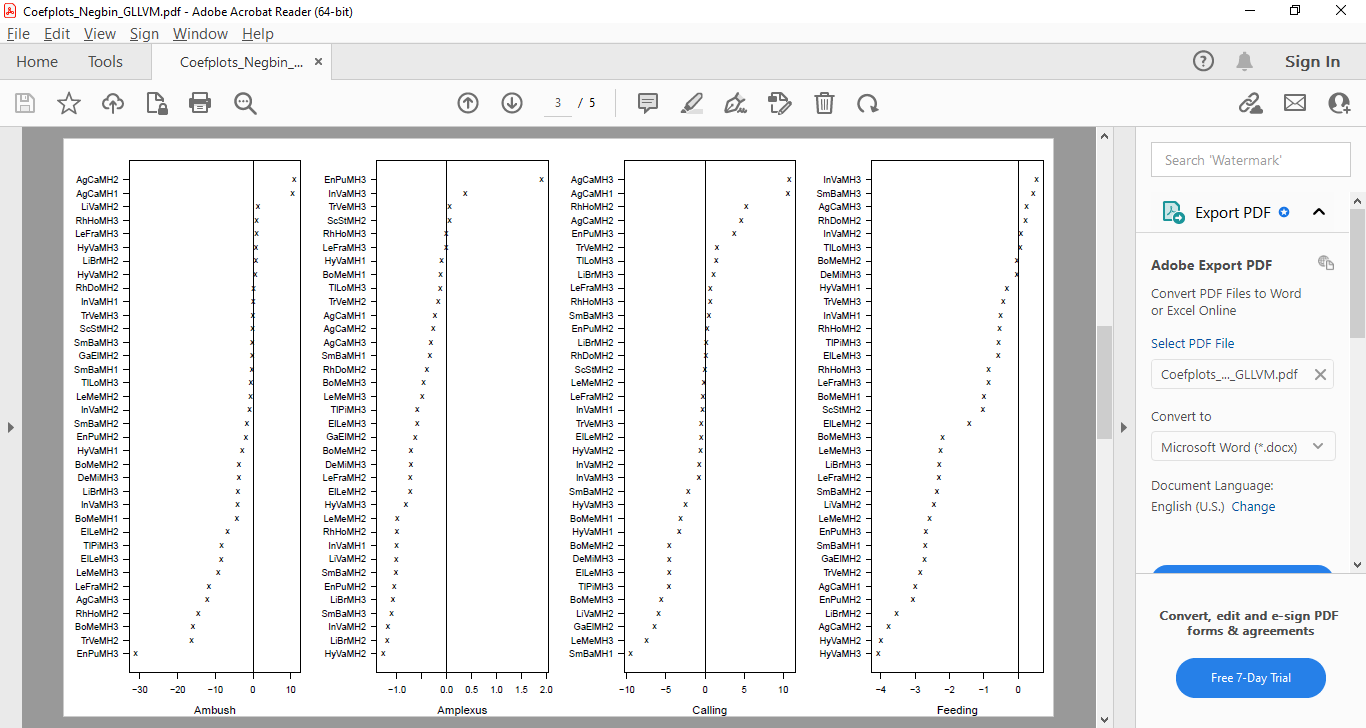

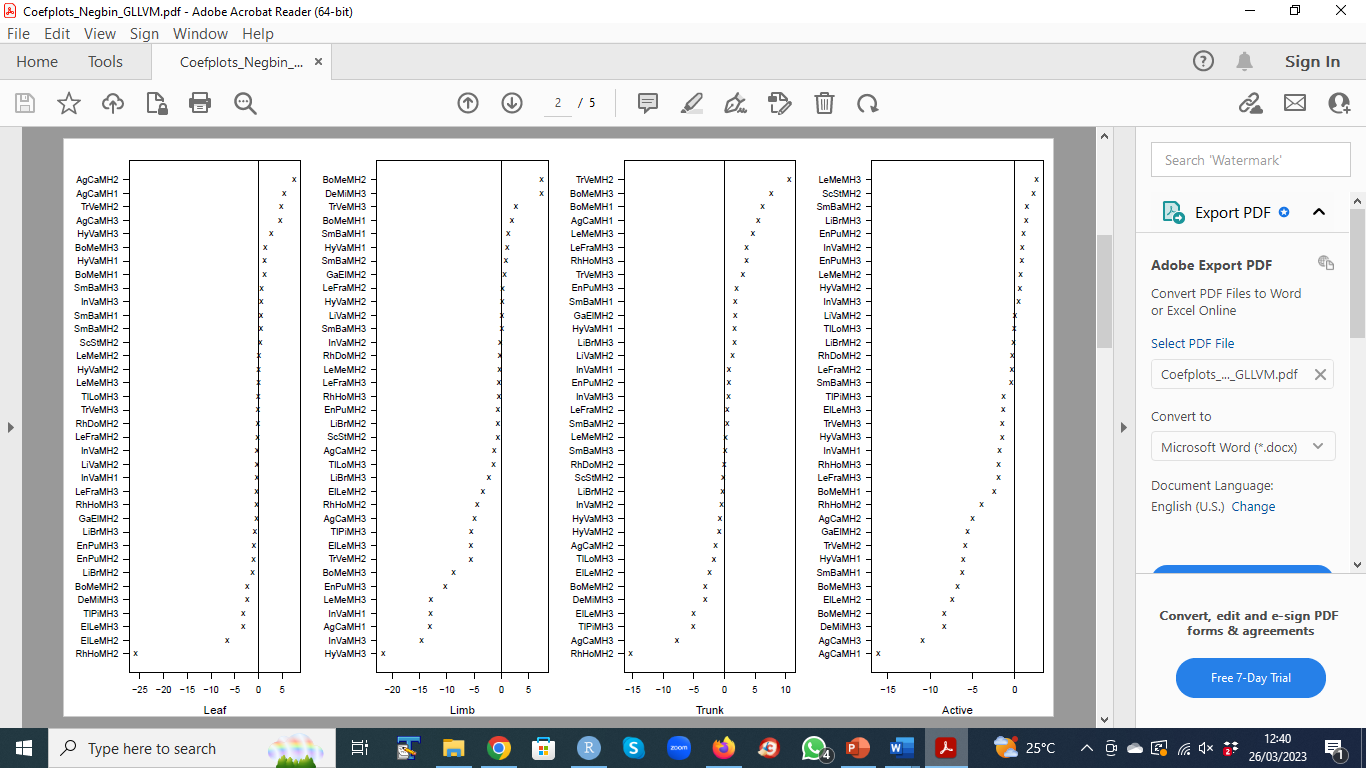

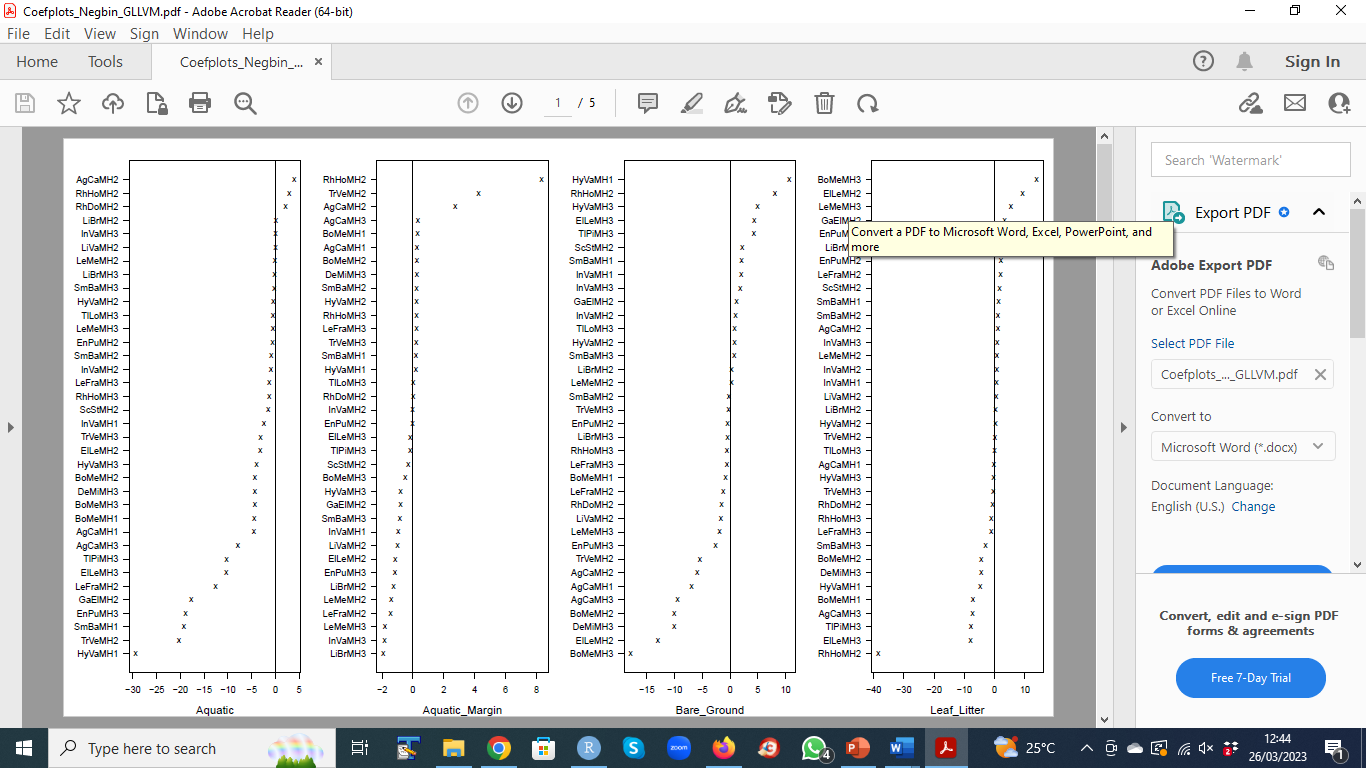


**Figure S.13:** Latent variable coefficient plots showing associations between amphibian species microhabitats and behaviours. Significant coefficients are identified by their confidence intervals not crossing zero. Those significant coefficients that are above zero show a positive association to the behaviours, those that are below zero show a negative association. Habitat codes: MH1 = Disturbed habitat; MH2 = Forest habitat; MH3 = Edge habitat. Species codes: AgCa = *Agalychnis callidryas*; BoMe = *Bolitoglossa mexicana*; DeMi = *Dendropsophus microcephalus*; ElLe = *Eleutherodactylus leprus*; EnPu = *Engystomops pustulosus*; GaEl = *Gastrophyryne elegans*; HyVa = *Hypopachus variolosus*; InVa = *Incilius valliceps*; LeFra = *Leptodactylus fragilis*; LeMe = *Leptodactylus melanonotus*; LiBr = *Lithobates brownorum*; LiVa = *Lithobates vaillanti*; RhDo = *Rhinophrynus dorsalis*; RhHo = *Rhinella horriblis*; ScSt = *Scinax staufferi*; SmBa = *Smilisca baudinii*; TlLo = *Tlalocohyla loquax*; TlPi = *Tlalocohyla picta*; TrPe = *Triprion petasatus*; TrVe = *Trachycephalus vermiculatus*.

**Figure S.14:** Latent variable coefficient plots showing associations between snake species, microhabitats and behaviours. Significant coefficients are identified by their confidence intervals not crossing zero. Those significant coefficients that are above zero show a positive association to the behaviours, those that are below zero show a negative association. Habitat codes: MH1 = Disturbed habitat; MH2 = Forest habitat; MH3 = Edge habitat. Species codes; AdQu = *Adelphicos quadrivigattum*, BoAs = *Bothrops asper*, BoIm = *Boa imperator*, ClSc = *Clelia scytalina*, CoBi = *Coniophanes bipunctatus*, CoIm = *Coniophanes imperialis*, CoSc = *Coniophanes schmidtii*, DrMa = *Drymobius margaritiferus*, GeSa = *Geophis sartorii*, ImCe = *Imantodes cenchoa*, LaAb = *Lampropeltis abnorma*, LeMex = *Leptophis mexicanu*s, LePr = *Leptophis praestans*, LeSe = *Leptodiera septentrionalis*, MaMen = *Masticophis mentovarius*, MaMe = *Mastigodryas melanonomus*, MiAp = *Micrurus apiatus,* NiDi = *Ninia diademata*, NiSe = *Ninia sebae*, OxKo = *Oxybelis koehleri*, OxPe = *Oxyrhopus petolarius*, PlEl = *Pliocercus elapoides*, PsFl = *Psuedelaphe flavirufa*, SiDi = *Sibon dimidiatus*, SiNe = *Sibon nebulatus*, SpPu = *Spilotes pullatus*, TaMo = *Tantilla moesta*, XeRa = *Xenodon rabdocephalus.*


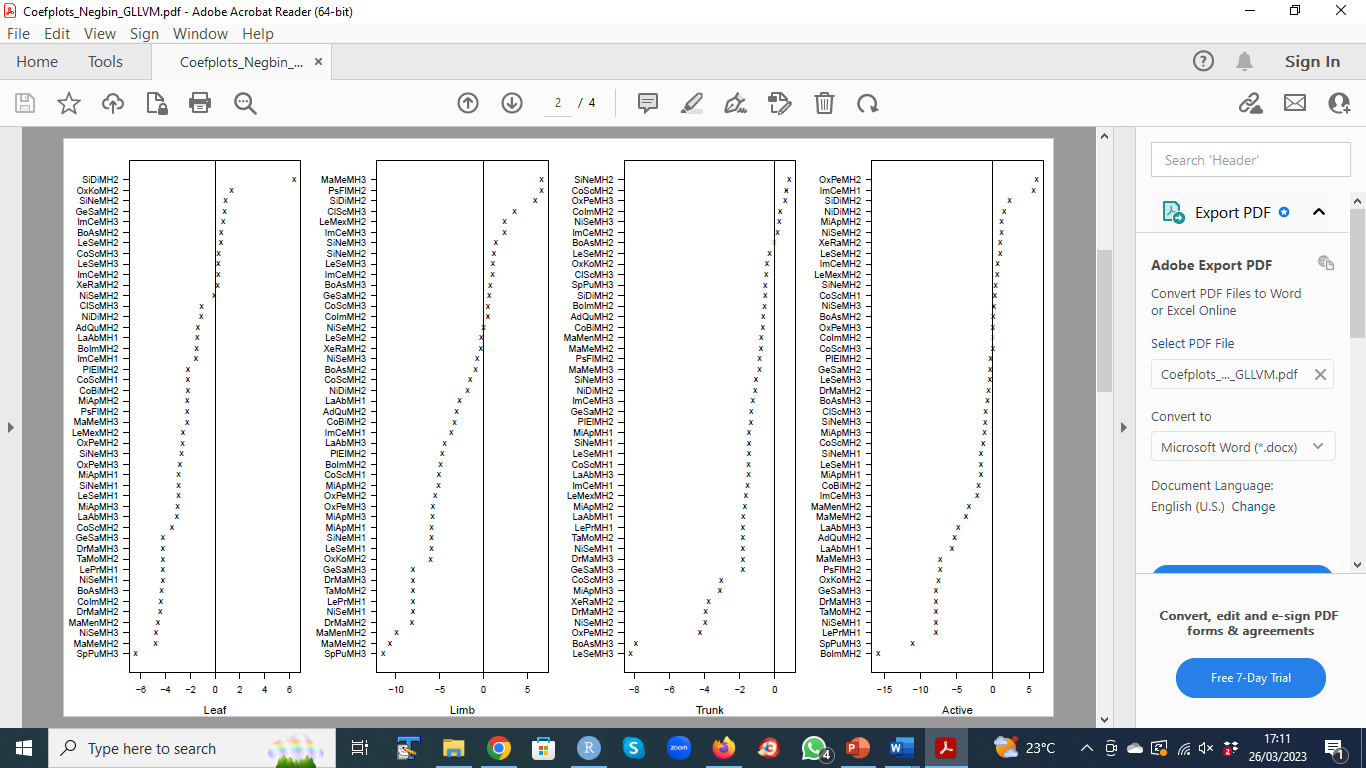

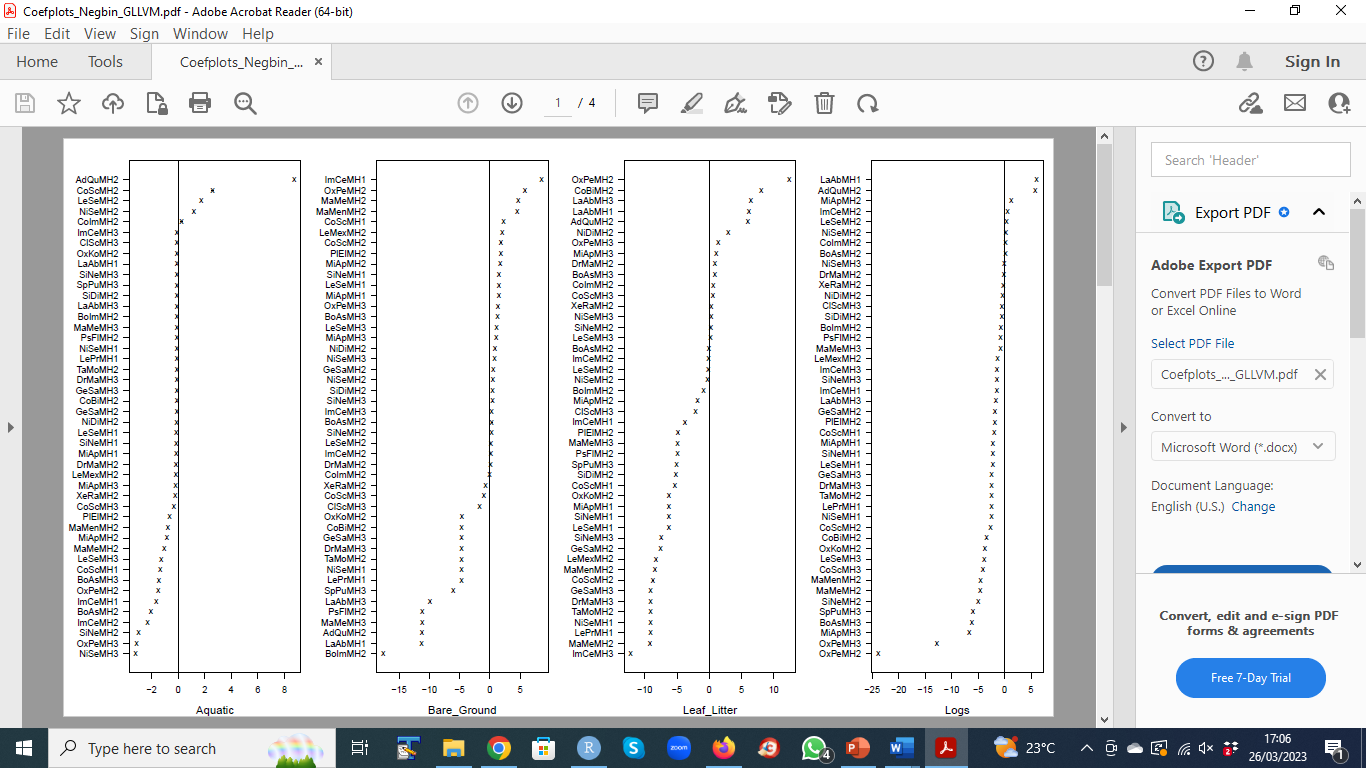

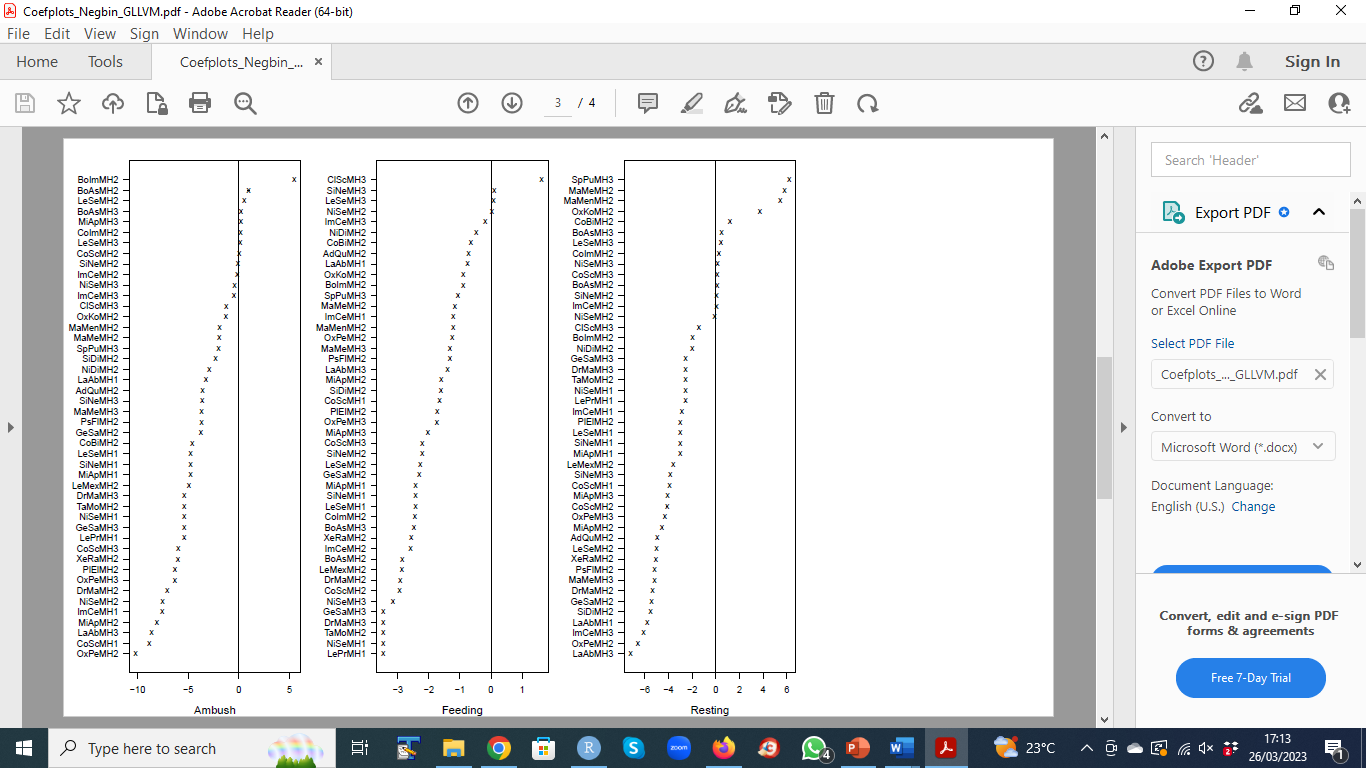


**Figure S.15:** Latent variable coefficient plots showing associations between lizard species, microhabitats and behaviours. Significant coefficients are identified by their confidence intervals not crossing zero. Those significant coefficients that are above zero show a positive association to the behaviours, those that are below zero show a negative association. Habitat codes: MH1 = Disturbed habitat; MH2 = Forest habitat; MH3 = Edge habitat. Species codes; BaVi = *Basiliscus vittatus*, CoCr = *Corytophanes cristatus*, CoEl = *Coleonyx elegans,* CoHe = *Corytophanes hernandesii*, HoFe = *Holcosus festiva*, HoUn = H*olcosus undulata*, MaUn = *Marisora brachypoda,* MeSc = *Mesoscincus schwartzei*, NoCa = *Norops capito*, NoLe = *Norops lemurinus*, NoRo = *Norops rodriguezii*, NoTr = *Norops tropidonotus*, NoUn = *Norops unilobatus*, ScCh = *Sceloporus chrysostictus*, ScTe = *Sceloporus teapensis*, ScChe = *Scincella cherriei,* SpGl = *Sphaerodactylus glaucus*, SpMi = *Sphaerodactylus millepunctatus*, ThRa = *Thecadactylus rapicauda*.


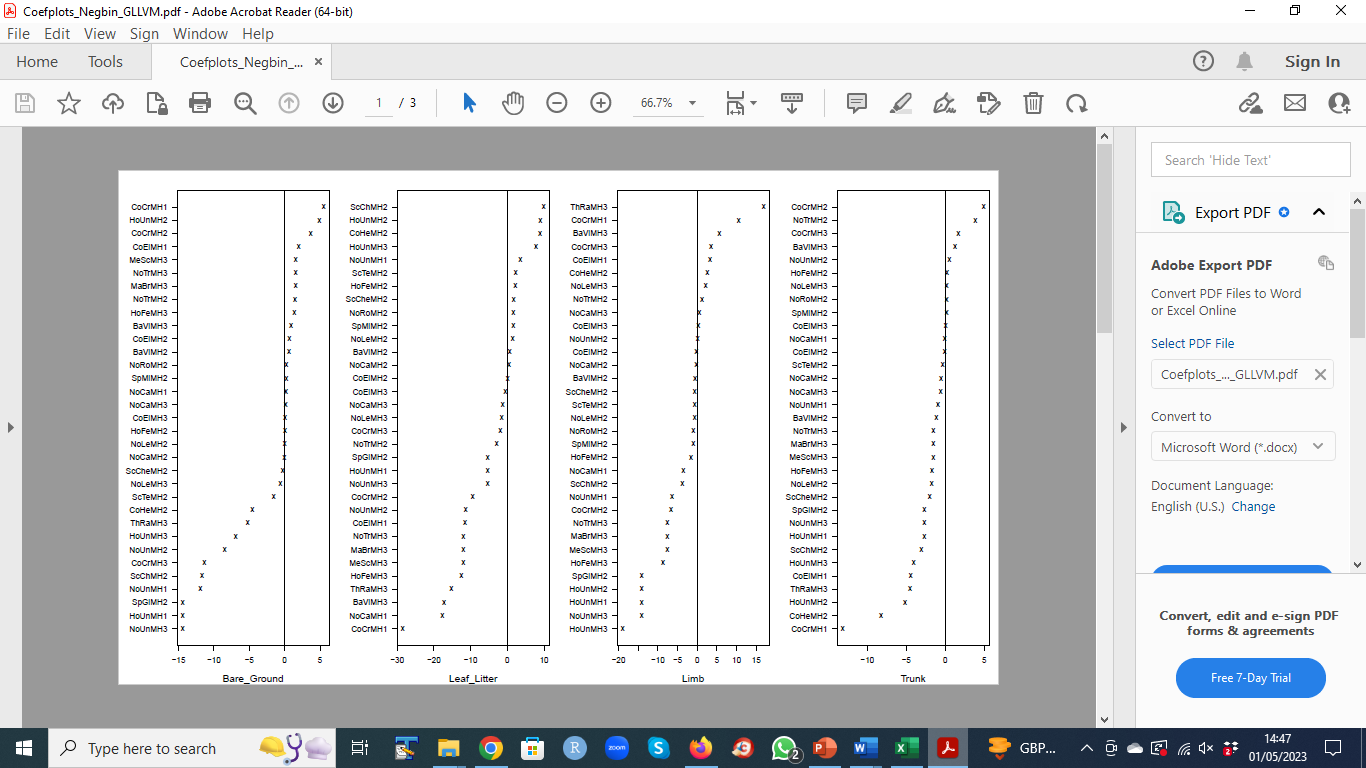

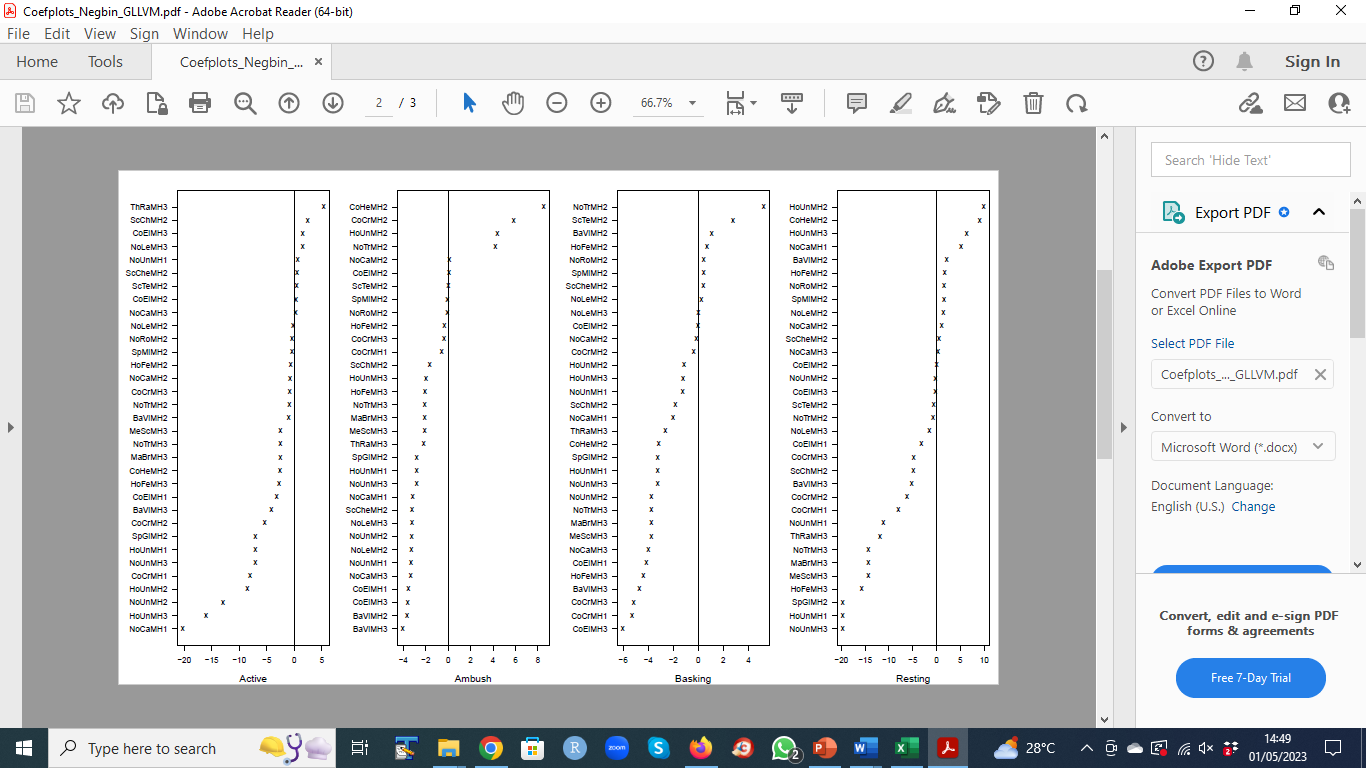

Supplement: Supplementary file 1 — Supplementary file1 (DOCX 9894 KB) [file 442_2025_5691_MOESM1_ESM.docx]
